# Supplementary material for: Cathepsin B-dependent glycolysis contributes to reduced renal uric acid excretion in hyperuricemia
Source: Commun Biol. 2025 Jun 2;8:845. doi: 10.1038/s42003-025-08303-5 (PMC12130491; doi:10.1038/s42003-025-08303-5)
Supplement: Supplementary file 2 — Supplementary Information [file 42003_2025_8303_MOESM2_ESM.pdf]

Supplemental Table S1. List of primer sequences used for qRT-PCR

Gene	Forward Primer	Reverse Primer
CTSB (Human)	5'-CTGCTGGCTGTAATGGTGGCTATC-3'	5'-GGGAGGGATGGAGTACGGTCTG-3'
β-actin (Human)	5'-GGCACCACACCTTCTACAATGAGC-3'	5'-GATAGCACAGCCTGGATAGCAACG-3'
CTSB (Mouse)	5'-ATAAGTGAGCTTTGGAGCGAGTTG-3'	5'-CCACAGTGTCTTCTCTAATCTCCTT-3'
β-actin (Mouse)	5'-CTACCTCATGAAGATCCTGACC-3'	5'-CACAGCTTCTCTTTGATGTCAC-3'

Supplementary Table S2. Characteristics of the control and the hyperuricemia group in male patients

Variables	All(n=24)	Control(n=9)	HUA(n=15)	*P* value
Age (years)	40.39±12.45	40.63±16.08	40.27±10.69	0.949
BMI (kg/m^2)	25.89±3.82	23.27±2.52	27.34±3.64	0.009**
Scr (μmol/L)	87.35±10.92	84.03±11.99	89.12±10.29	0.297
eGFR(ml/min/1.73m^2)	96.91±17.19	99.25±16.49	95.67±17.98	0.645
SUA (μmol/L)	471.43±112.65	358.13±49.00	531.87±86.55	<0.001***
Ucr (mmol)	18.78±8.22	20.97±12.50	17.46±4.10	0.436
UUA (mmol)	4.43±1.24	4.59±1.25	4.34±1.27	0.631
FEua (%)	4.74±1.61	5.57±1.83	4.30±1.33	0.006**
24-hCTSB (ng)	12.55±6.08	8.12±2.99	14.91±6.03	0.002**

***P* < 0.01, *** *P* < 0.001

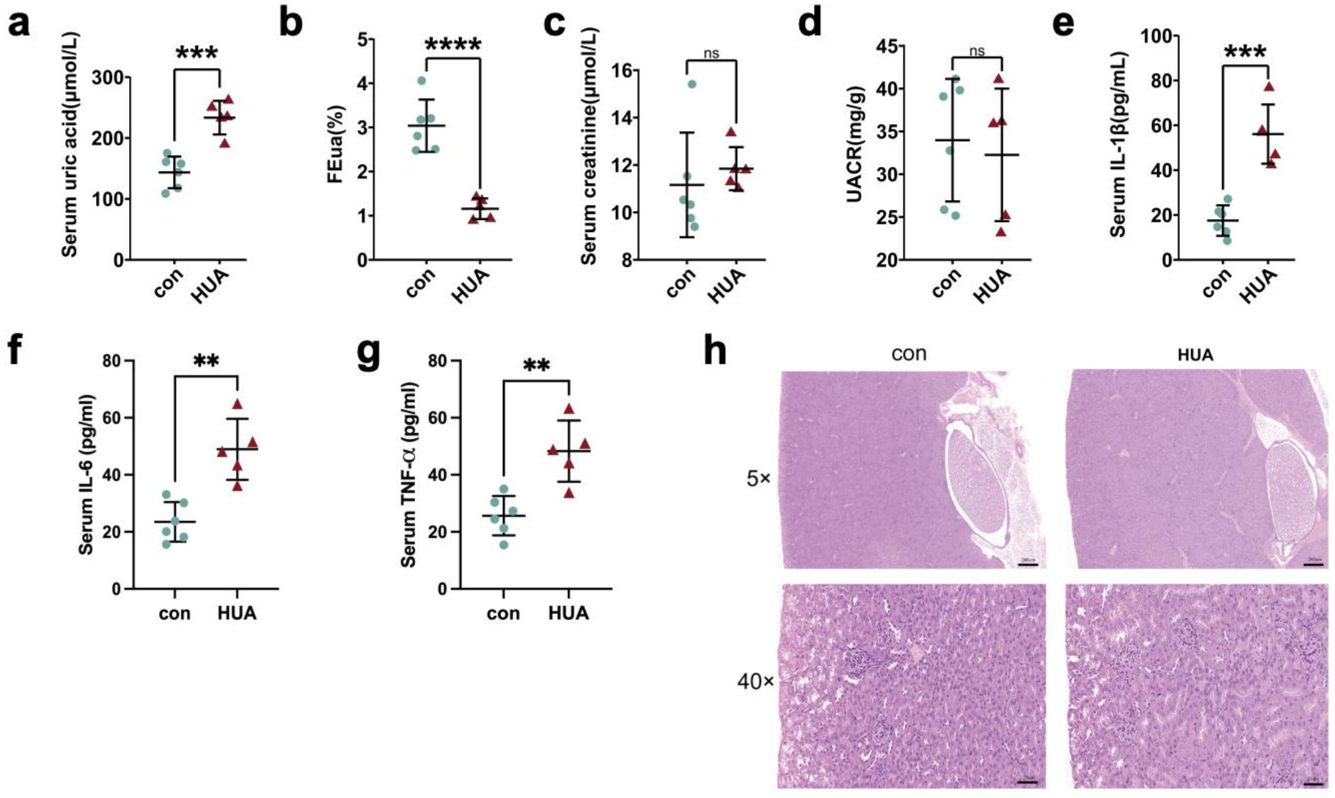

Supplementary Figure S1. Establishment of HUA mice. The level of (a) SUA, (b) FEua, (c) Scr, (d) U-MALB, (e) IL-1β, (f) IL-6 and (g) TNF-α in control and HUA mice. (h) HE staining of the kidneys incontrol and HUA mice. Bars = 25 μm (40×). Bars = 200 μm (5×). n ≥ 3. ** $P < 0.01$, *** $P < 0.001$, **** $P < 0.0001$.

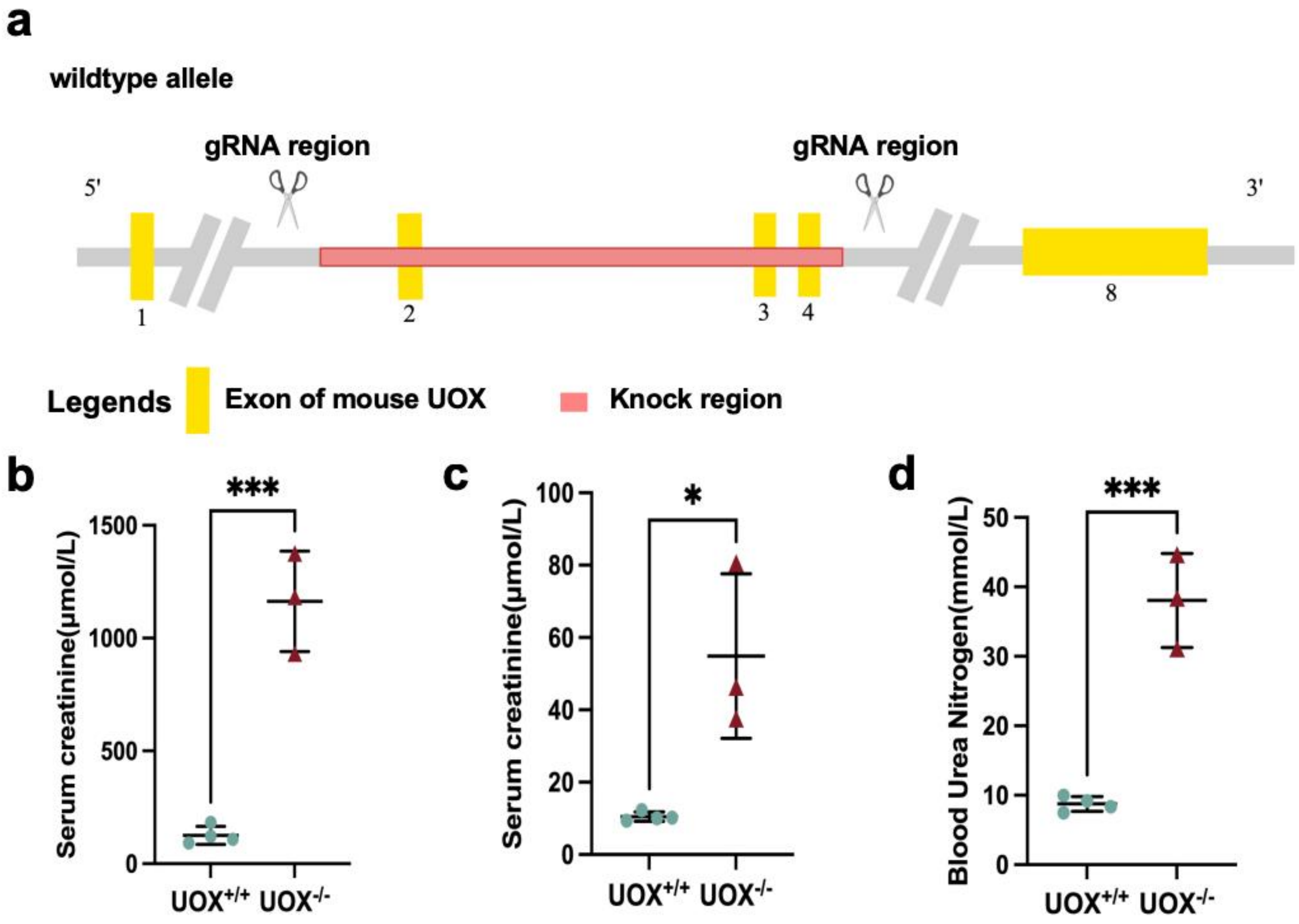

Supplementary Figure S2. Construction and verification of uricase knockout mice. (a) Construction scheme of uricase knockout mice. The level of (b) SUA (c) Scr and (d) BUN in uricase-knockout HUA mice. n ≥ 3. * $P < 0.05$, *** $P < 0.001$.

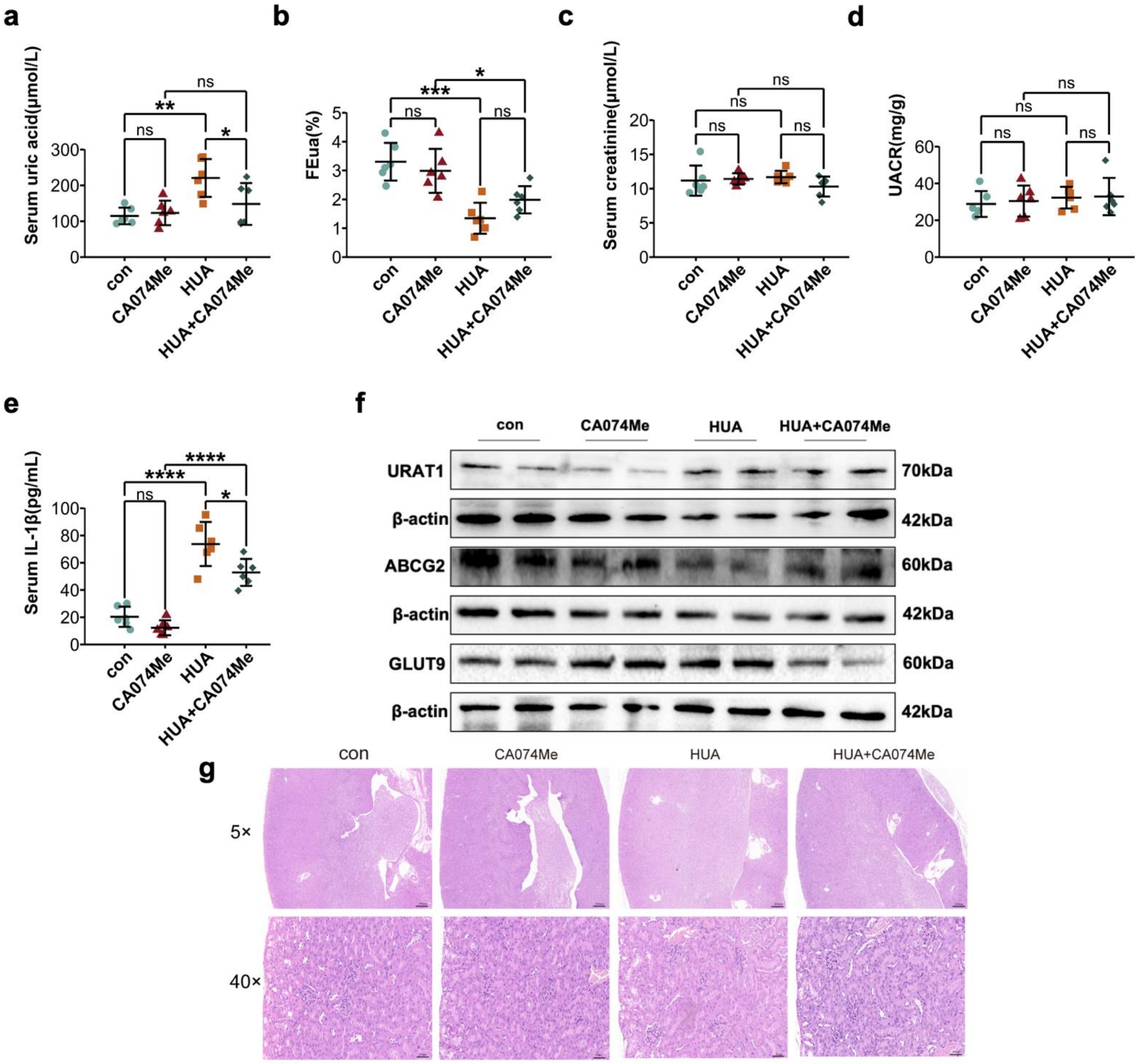

Supplementary Figure S3. Inhibiting CTSB reduced inflammation and increased renal uric acid excretion in vivo. Levels of (a) SUA, (b) FEua, (c) Scr, (d)U-MALB and (e)IL-1β in different groups of mice. (f) Western blot of URAT1, ABCG2 and GLUT9 in kidneys of different groups of mice. (g) HE staining of kidney in different groups of mice. Bars = 25μm(40×), Bars = 200μm(5×). n ≥ 3. * $P < 0.05$, ** $P < 0.01$, *** $P < 0.001$, **** $P < 0.0001$.

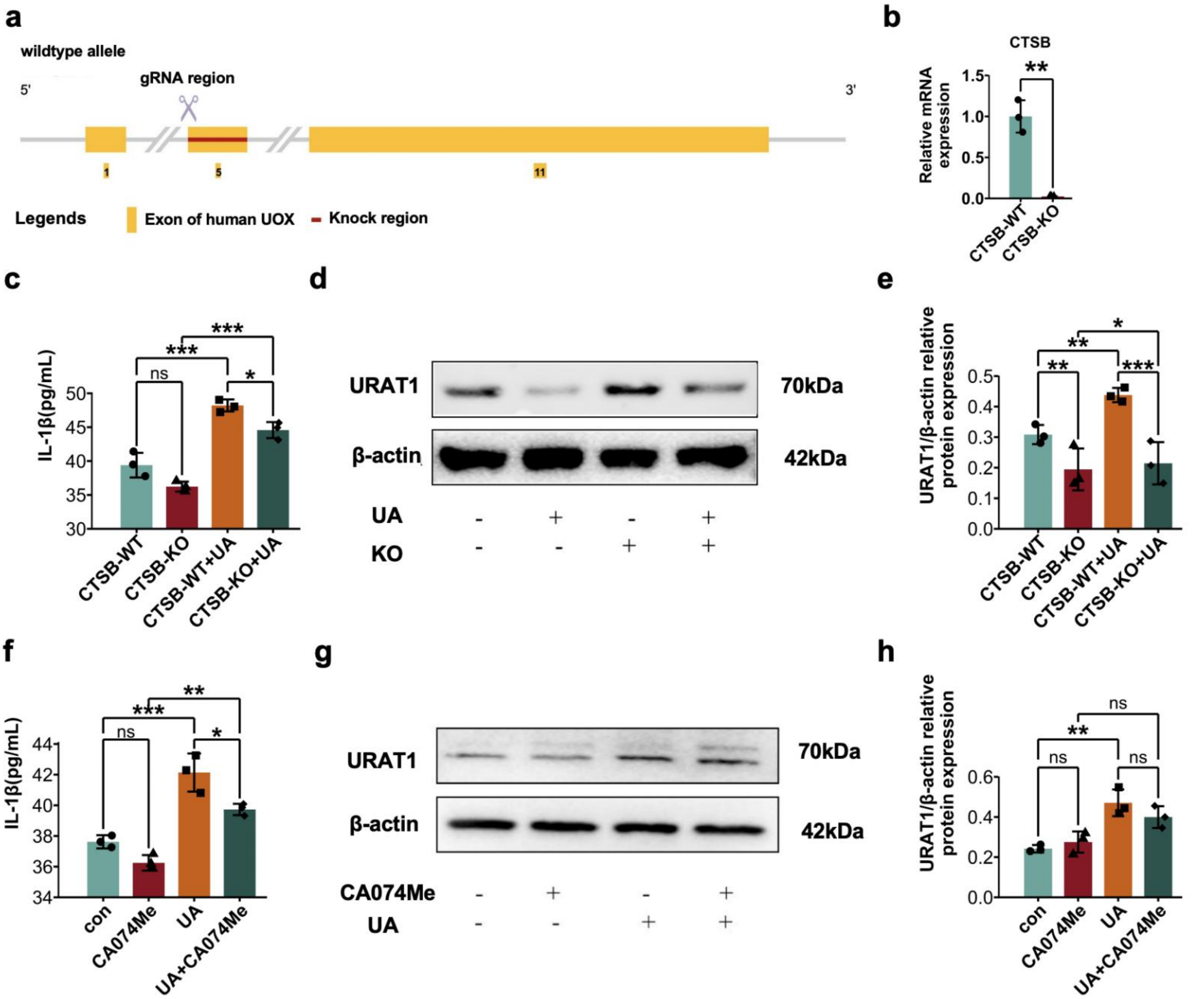

Supplementary Figure S4. Inhibiting or knocking out of CTSB reduced inflammation and increased renal uric acid excretion in vitro. (a) CTSB knockout protocol in renal proximal tubular epithelial cells. (b) Validation of CTSB knockout in renal proximal tubular epithelial cells. (c) The levels of IL-1β after knockout of CTSB in vitro. (d) Western blot of URAT1 in CTSB-KO cells. (e) Statistical analysis of protein expression. (f) The levels of IL-1β after treated with CA074Me in vitro. (g) Western blot of URAT1 in HK2 cells treated with CA074Me.. (h) Statistical analysis of protein expression. * $P < 0.05$, ** $P < 0.01$. *** $P < 0.001$, **** $P < 0.0001$.

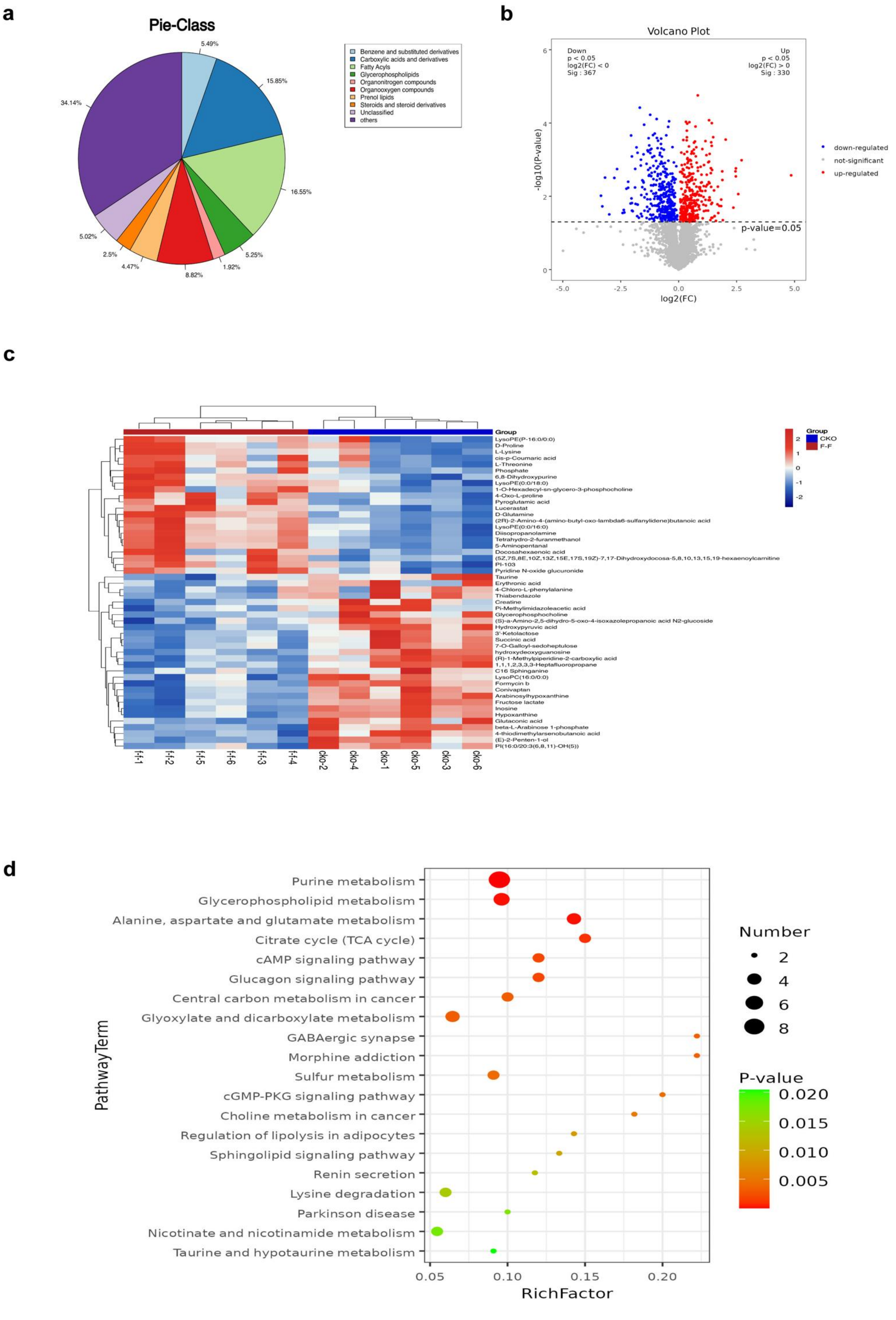
a
Pie-Class
5.49%
15.85%
16.55%
5.25%
1.92%
8.82%
4.47%
2.5%
5.02%
34.14%
Benzene and substituted derivatives
Carboxylic acids and derivatives
Fatty Acyls
Glycerophospholipids
Organonitrogen compounds
Organooxygen compounds
Prenol lipids
Steroids and steroid derivatives
Unclassified
others
b
Volcano Plot
Down
p < 0.05
log2(FC) < 0
Sig : 367
Up
p < 0.05
log2(FC) > 0
Sig : 330
-log10(P-value)
log2(FC)
p-value=0.05
down-regulated
not-significant
up-regulated
c
Group
LysoPE(P-16:0/0:0)
D-Proline
L-Lysine
cis-p-Coumaric acid
L-Threonine
Phosphate
6,8-Dihydroxypurine
LysoPE(0:0/18:0)
1-O-Hexadecyl-sn-glycero-3-phosphocholine
4-Oxo-L-proline
Pyroglutamic acid
Lucerastat
D-Glutamine
(2R)-2-Amino-4-(amino-butyl-oxo-lambda6-sulfanylidene)butanoic acid
LysoPE(0:0/16:0)
Diisopropanolamine
Tetrahydro-2-furanmethanol
5-Aminopentanal
Docosahexaenoic acid
(5Z,7S,8E,10Z,13Z,15E,17S,19Z)-7,17-Dihydroxydocosa-5,8,10,13,15,19-hexaenoylcarnitine
PI-103
Pyridine N-oxide glucuronide
Taurine
Erythronic acid
4-Chloro-L-phenylalanine
Thiabendazole
Creatine
Pi-Methylimidazoleacetic acid
Glycerophosphocholine
(S)-a-Amino-2,5-dihydro-5-oxo-4-isoxazolepropanoic acid N2-glucoside
Hydroxypyruvic acid
3'-Ketolactose
Succinic acid
7-O-Galloyl-sedoheptulose
hydroxydeoxyguanosine
(R)-1-Methylpiperidine-2-carboxylic acid
1,1,1,2,3,3,3-Heptafluoropropane
C16 Sphinganine
LysoPC(16:0/0:0)
Formycin b
Conivaptan
Arabinosylhypoxanthine
Fructose lactate
Inosine
Hypoxanthine
Glutaconic acid
beta-L-Arabinose 1-phosphate
4-thiodimethylarsenobutanoic acid
(E)-2-Penten-1-ol
PI(16:0/20:3(6,8,11)-OH(5))
f-f-1
f-f-2
f-f-5
f-f-6
f-f-3
f-f-4
cko-2
cko-4
cko-1
cko-5
cko-3
cko-6
Group
CKO
F-F
d
Purine metabolism
Glycerophospholipid metabolism
Alanine, aspartate and glutamate metabolism
Citrate cycle (TCA cycle)
cAMP signaling pathway
Glucagon signaling pathway
Central carbon metabolism in cancer
Glyoxylate and dicarboxylate metabolism
GABAergic synapse
Morphine addiction
Sulfur metabolism
cGMP-PKG signaling pathway
Choline metabolism in cancer
Regulation of lipolysis in adipocytes
Sphingolipid signaling pathway
Renin secretion
Lysine degradation
Parkinson disease
Nicotinate and nicotinamide metabolism
Taurine and hypotaurine metabolism
PathwayTerm
RichFactor
Number
P-value

Supplementary Figure S5. Renal metabolism was affected after CTSB knockout in renal proximal tubular epithelial cells in mice. (a) Classification of detected metabolites. (b) Volcanic maps of differential metabolites between the two groups of mice kidneys. (c) Heat map of TOP50 differential metabolites in two groups of mice kidneys. (d) KEGG enrichment analysis of differential metabolites. n = 6.

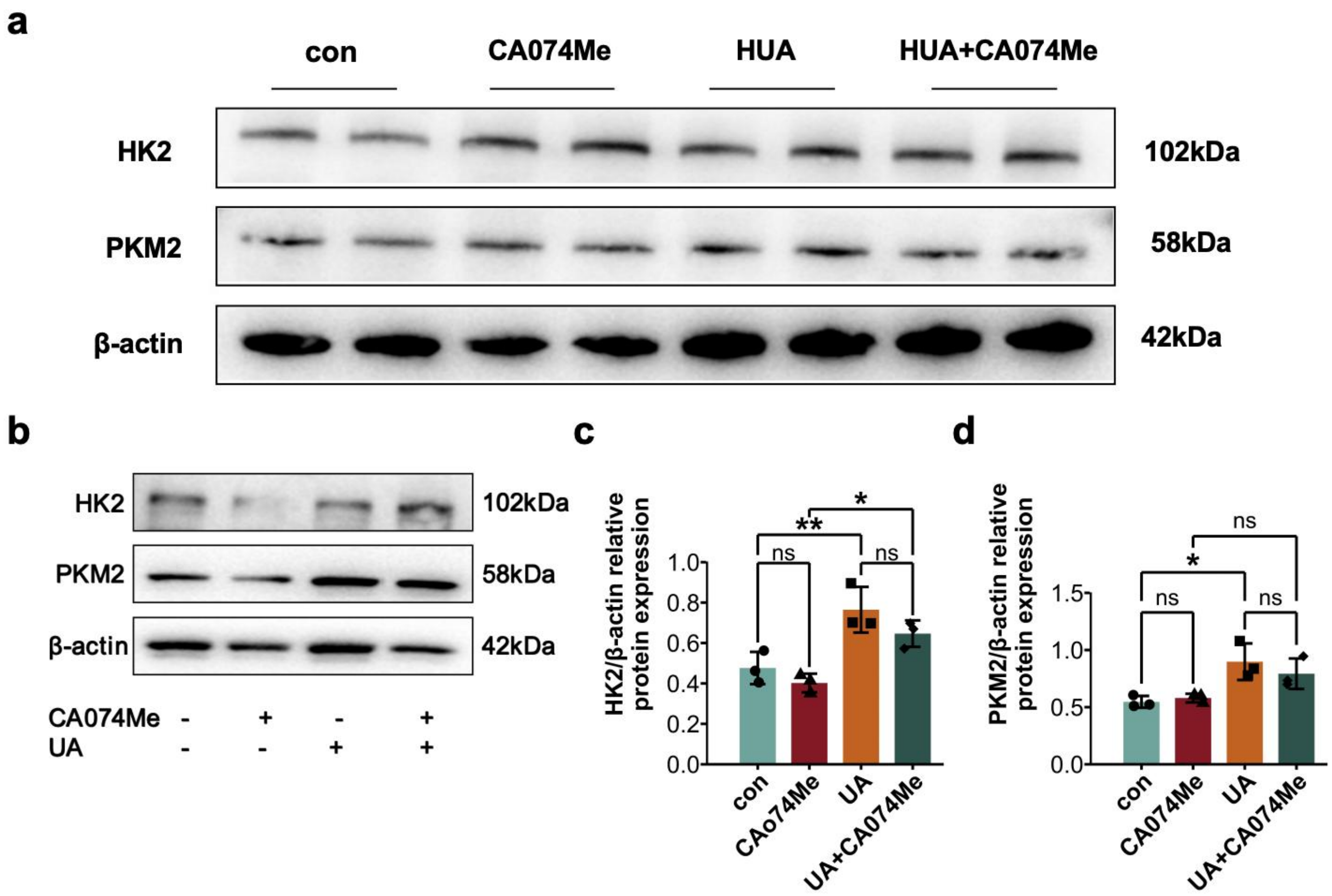

Supplementary Figure S6. Glycolysis reduced when CTSB was inhibited in vivo and in vitro. (a) Western blot of HK2 and PKM2 in kidneys after CTSB was inhibited in mice. (b) The protein expression of HK2 and PKM2 after CTSB inhibition in vitro. Statistical analysis of (c) HK2 and (d) PKM2 protein expression. n ≥ 3. * $P < 0.05$, ** $P < 0.01$.

figure 1

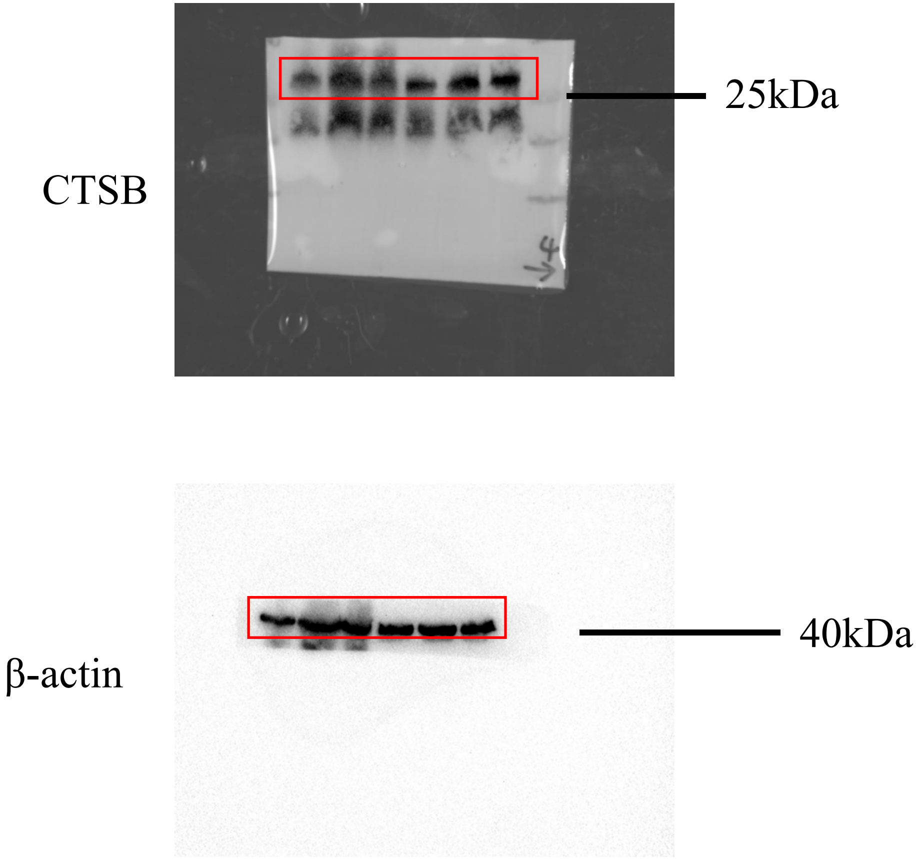

Fig.1e

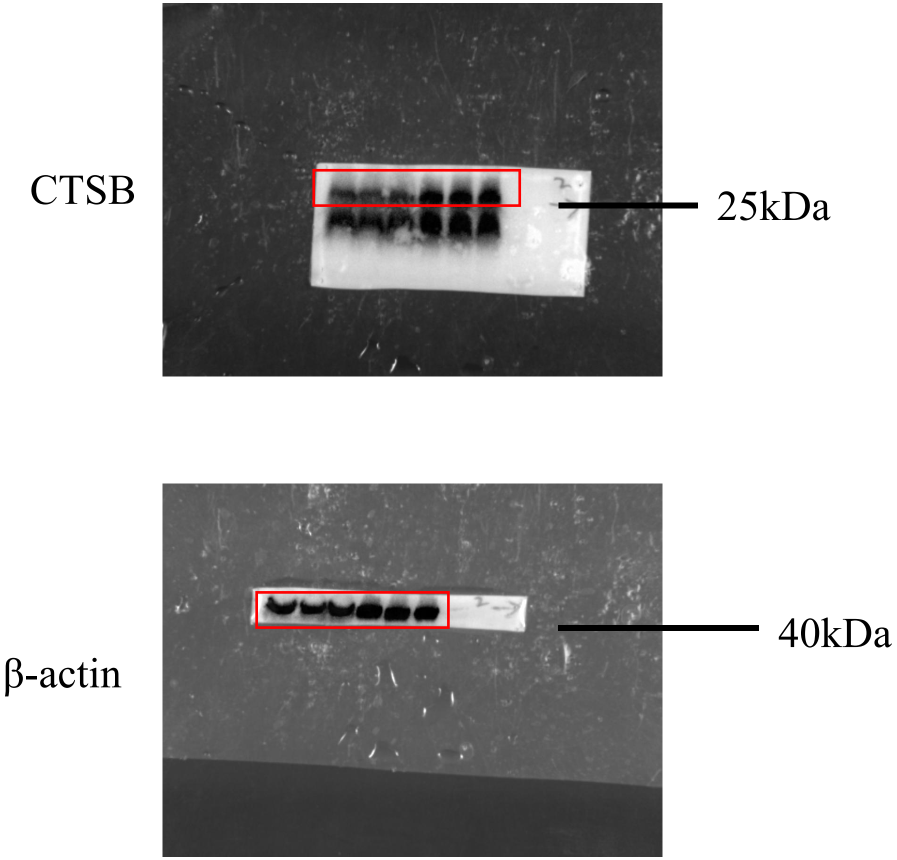

Fig.1h

figure 1

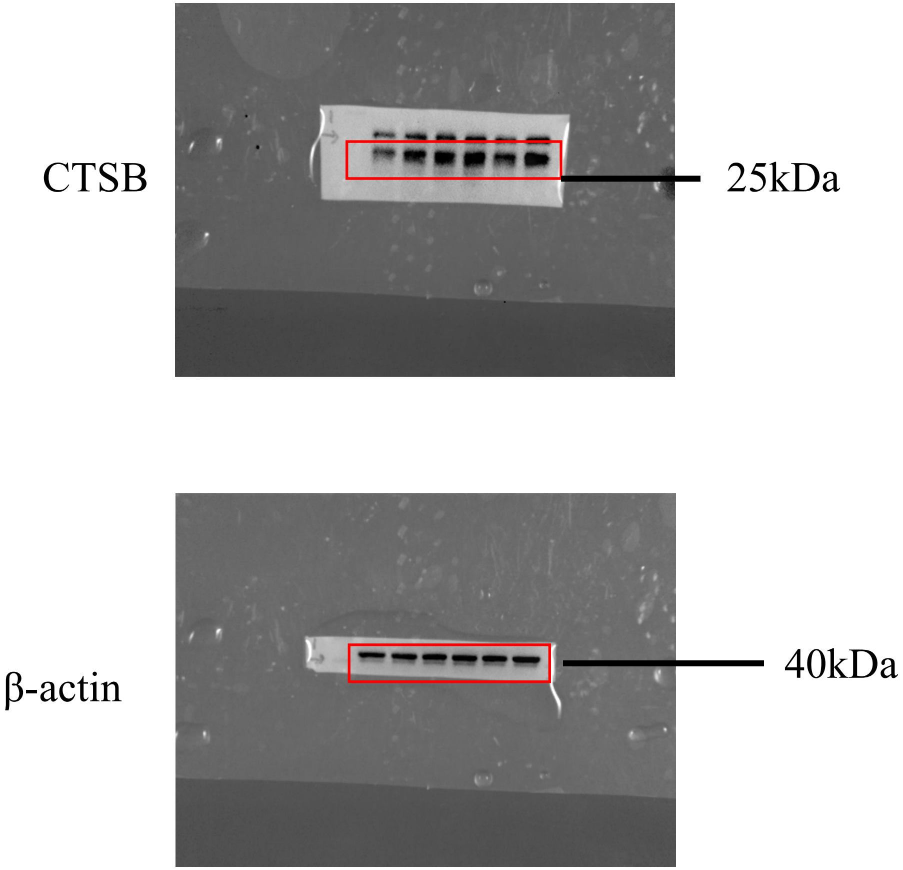

Fig.1m

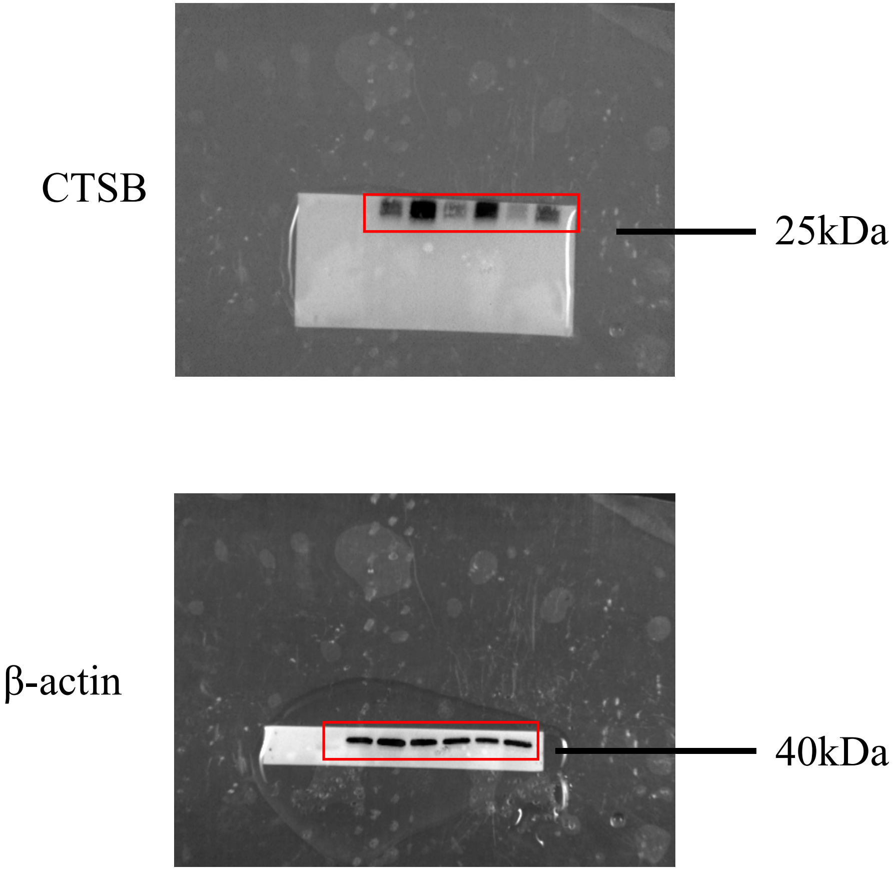

Fig.1q

figure 2

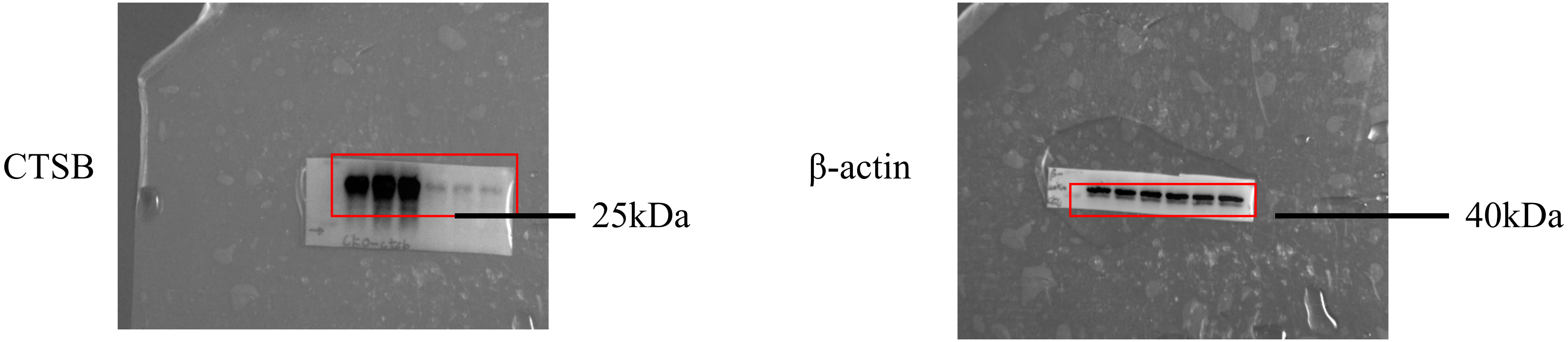

Fig.2b

figure 2

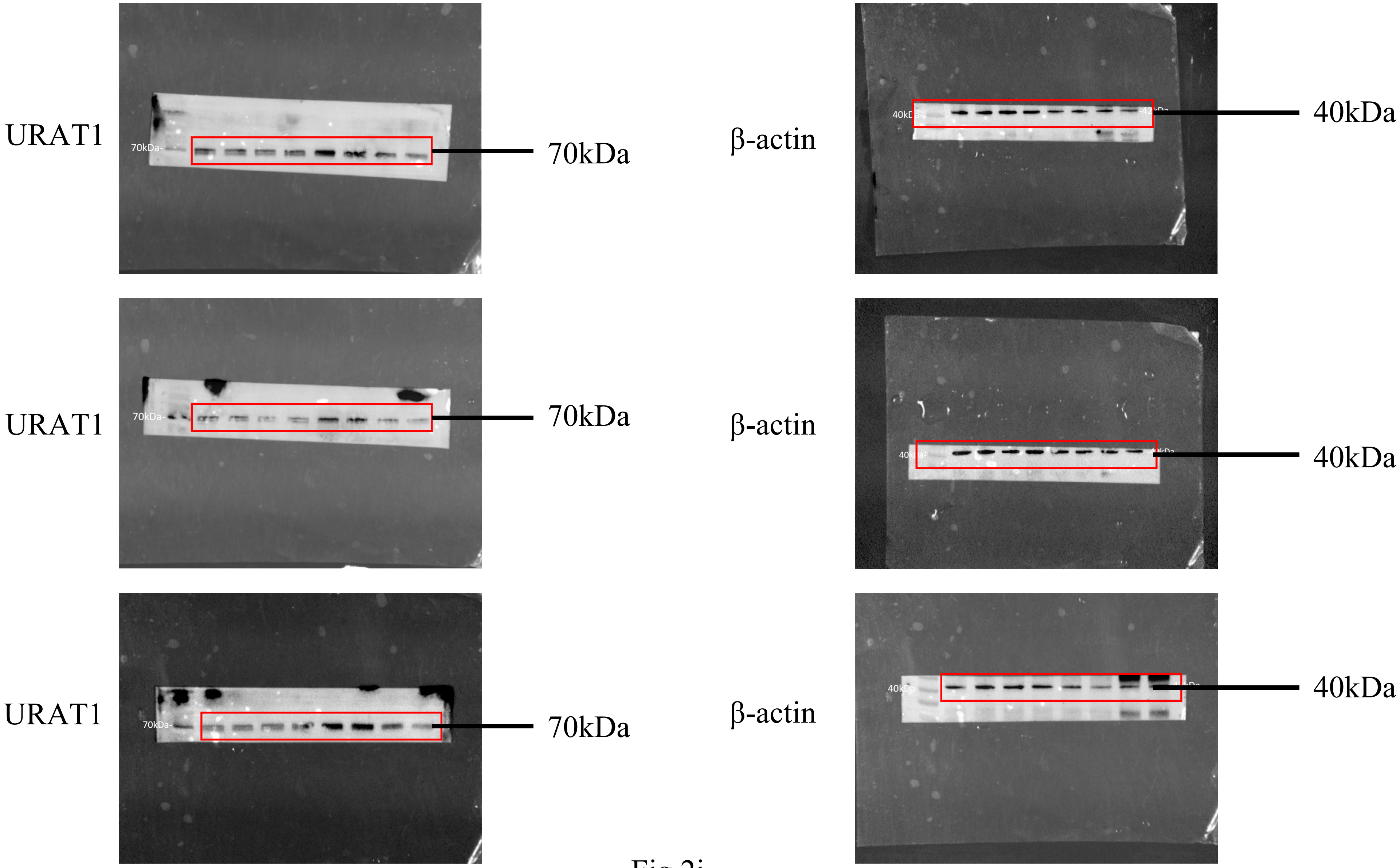

Fig.2j

figure 2

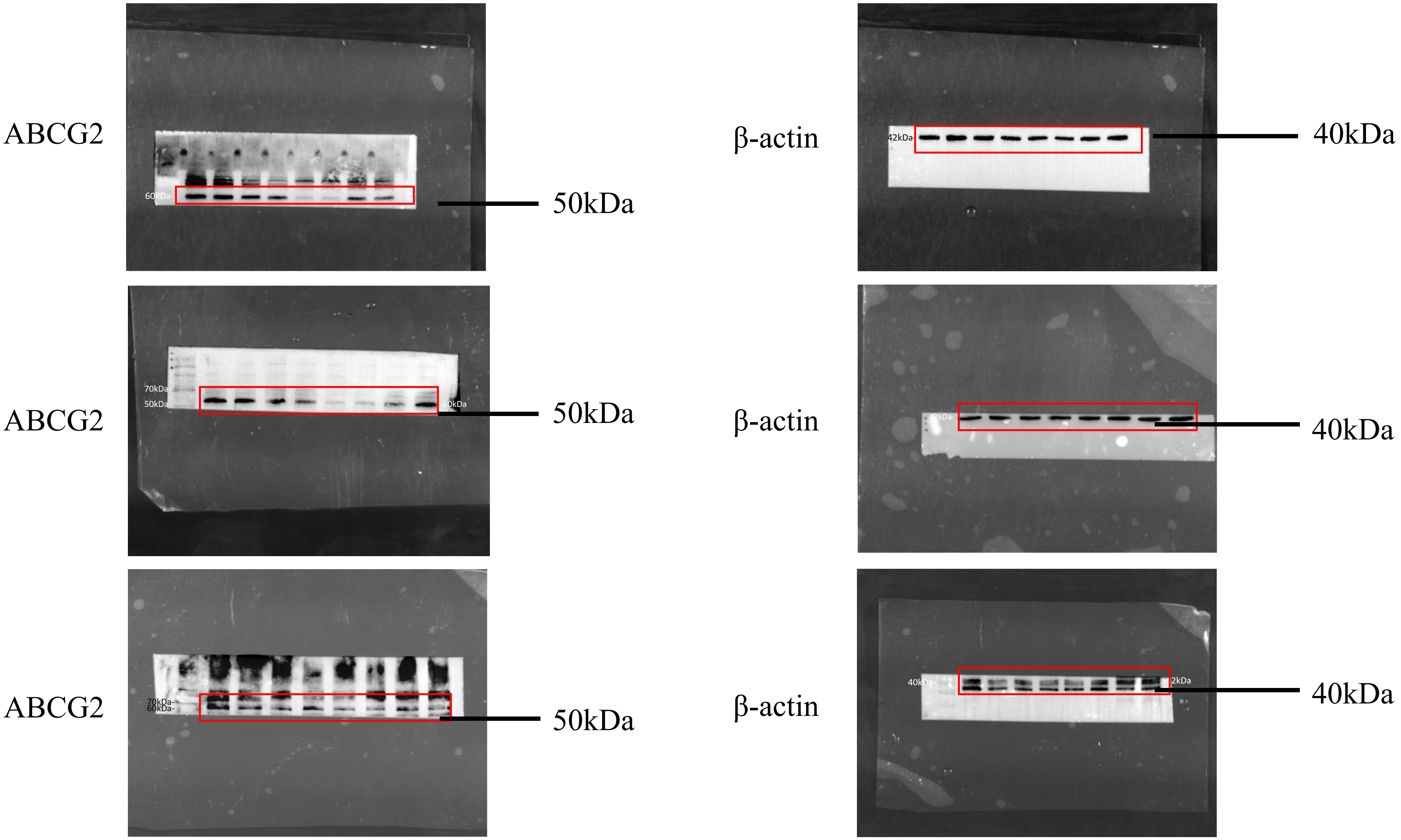

Fig.2j

figure 2

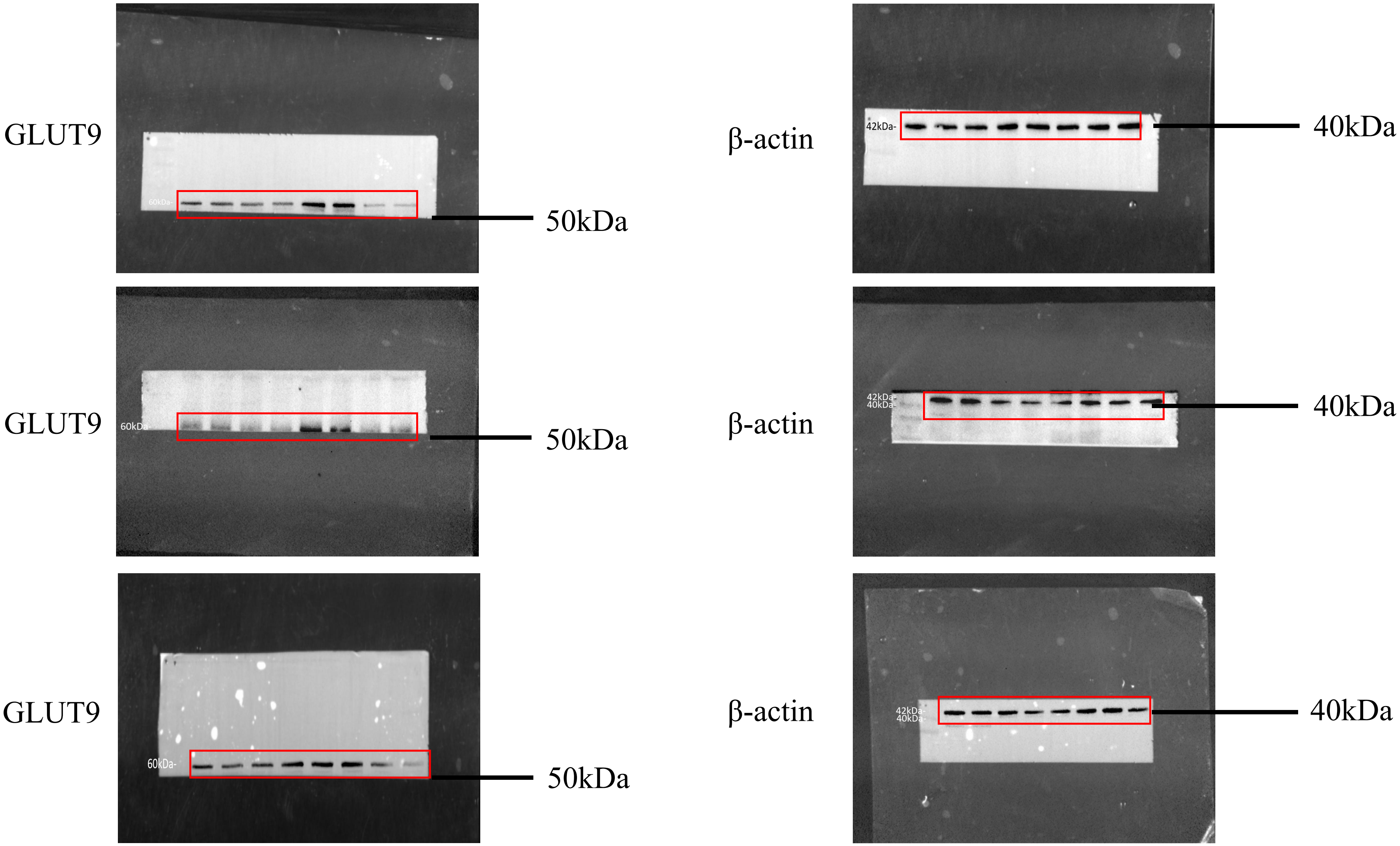

Fig.2j

figure 4

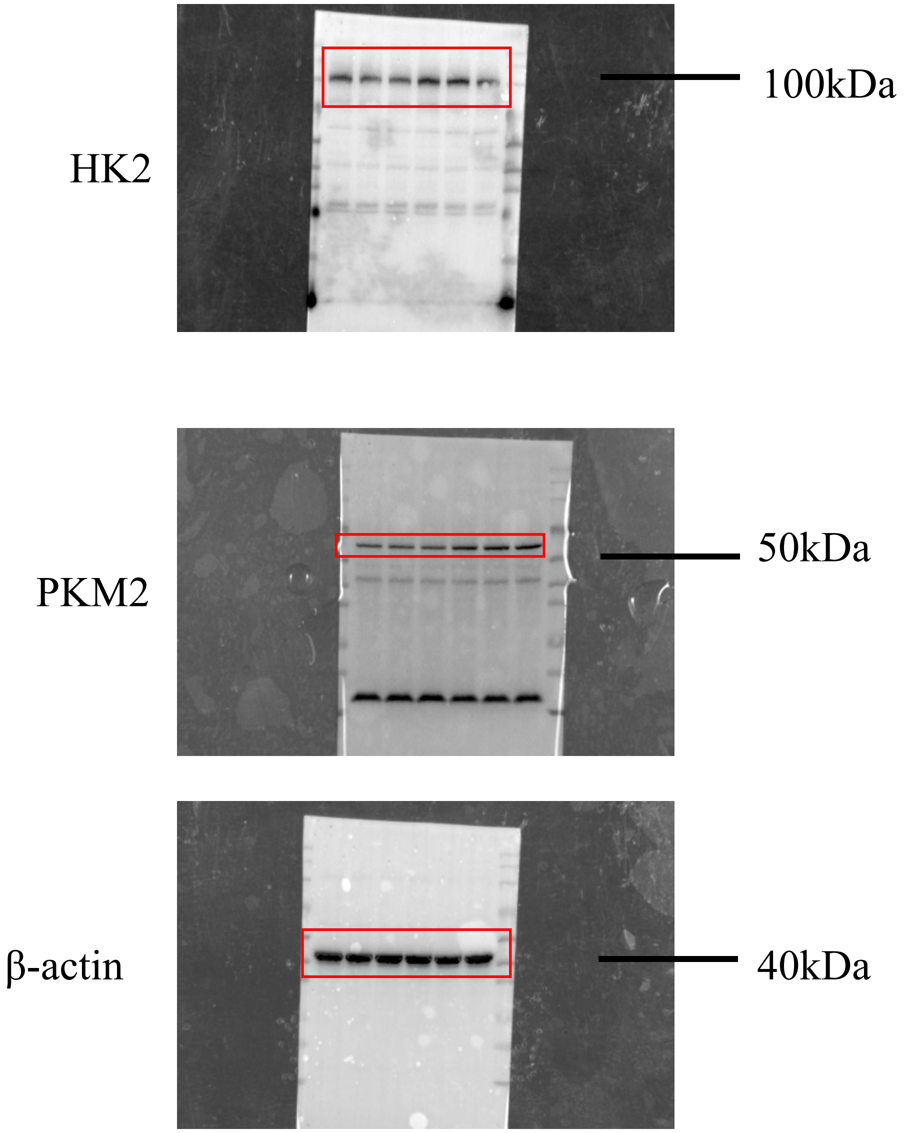

Fig.4a

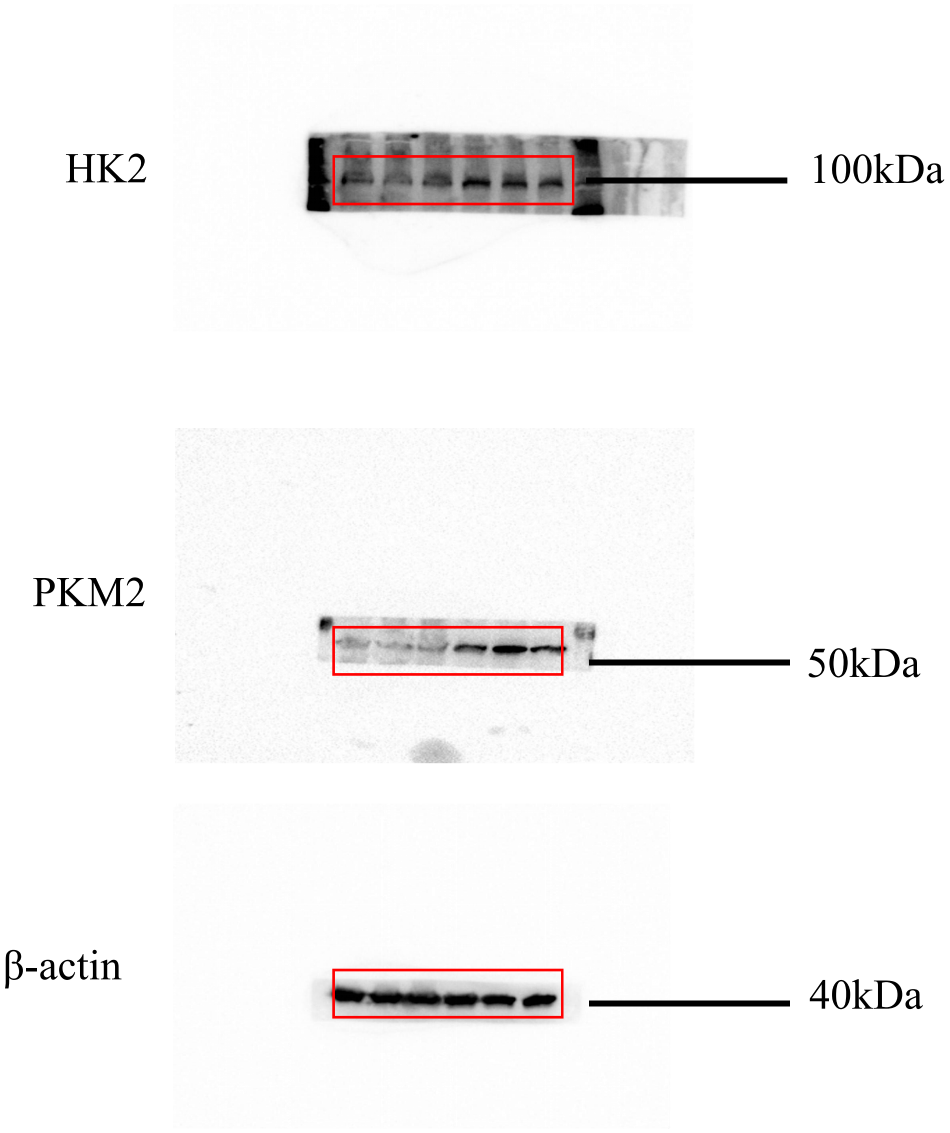

Fig.4f

figure 5

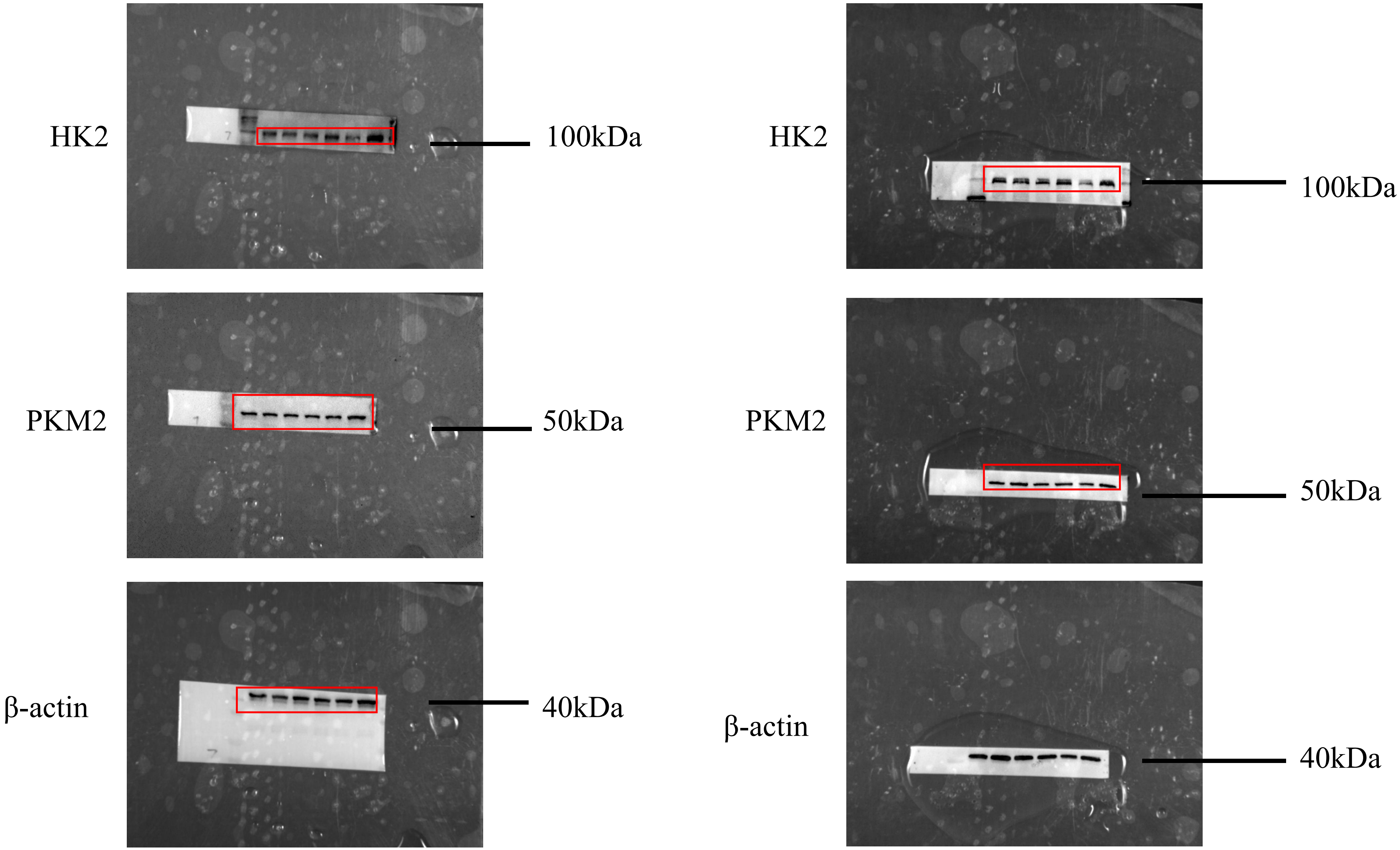

Fig.5a Fig.5d

figure 6

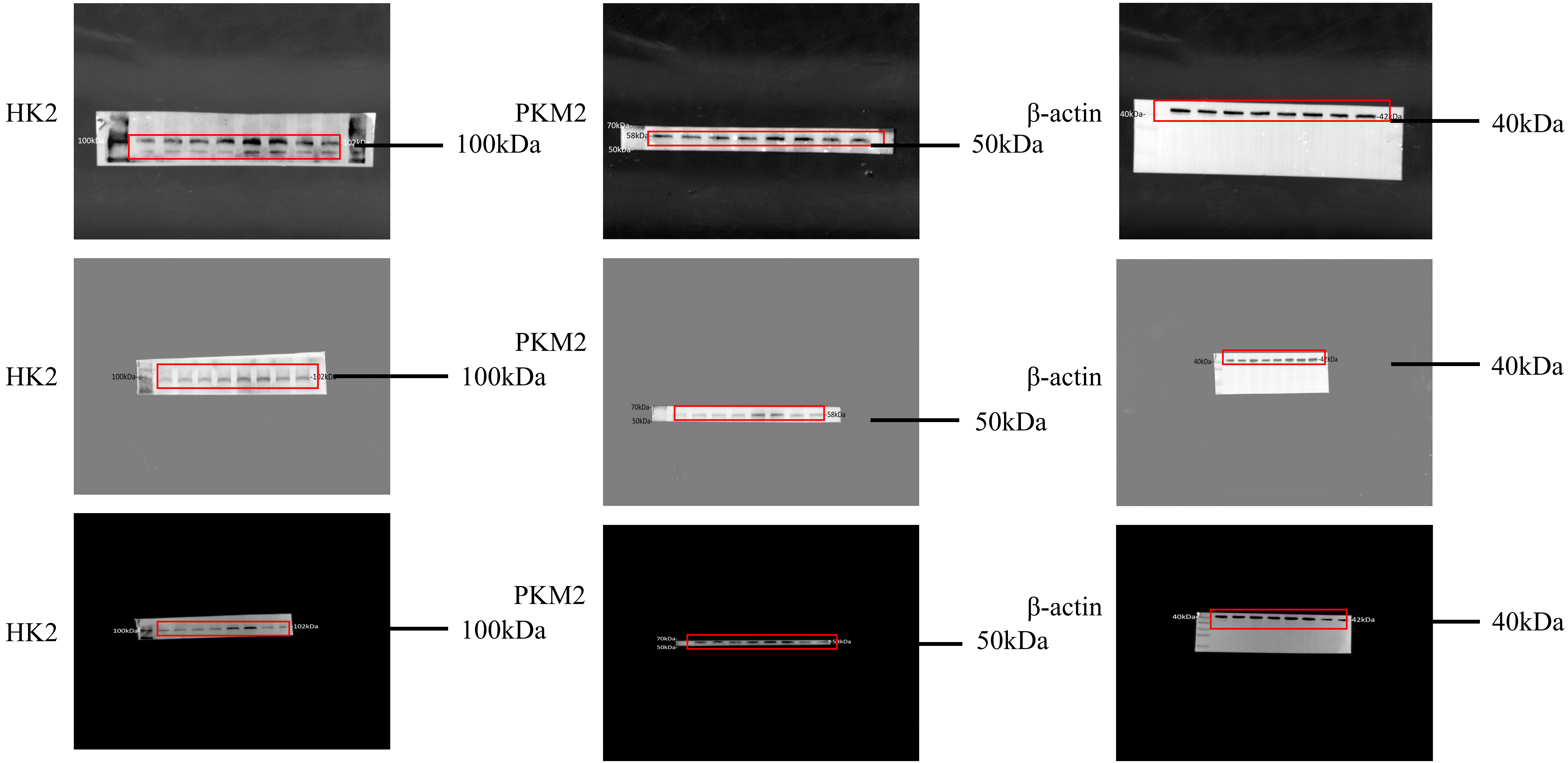

Fig.6c

figure 6

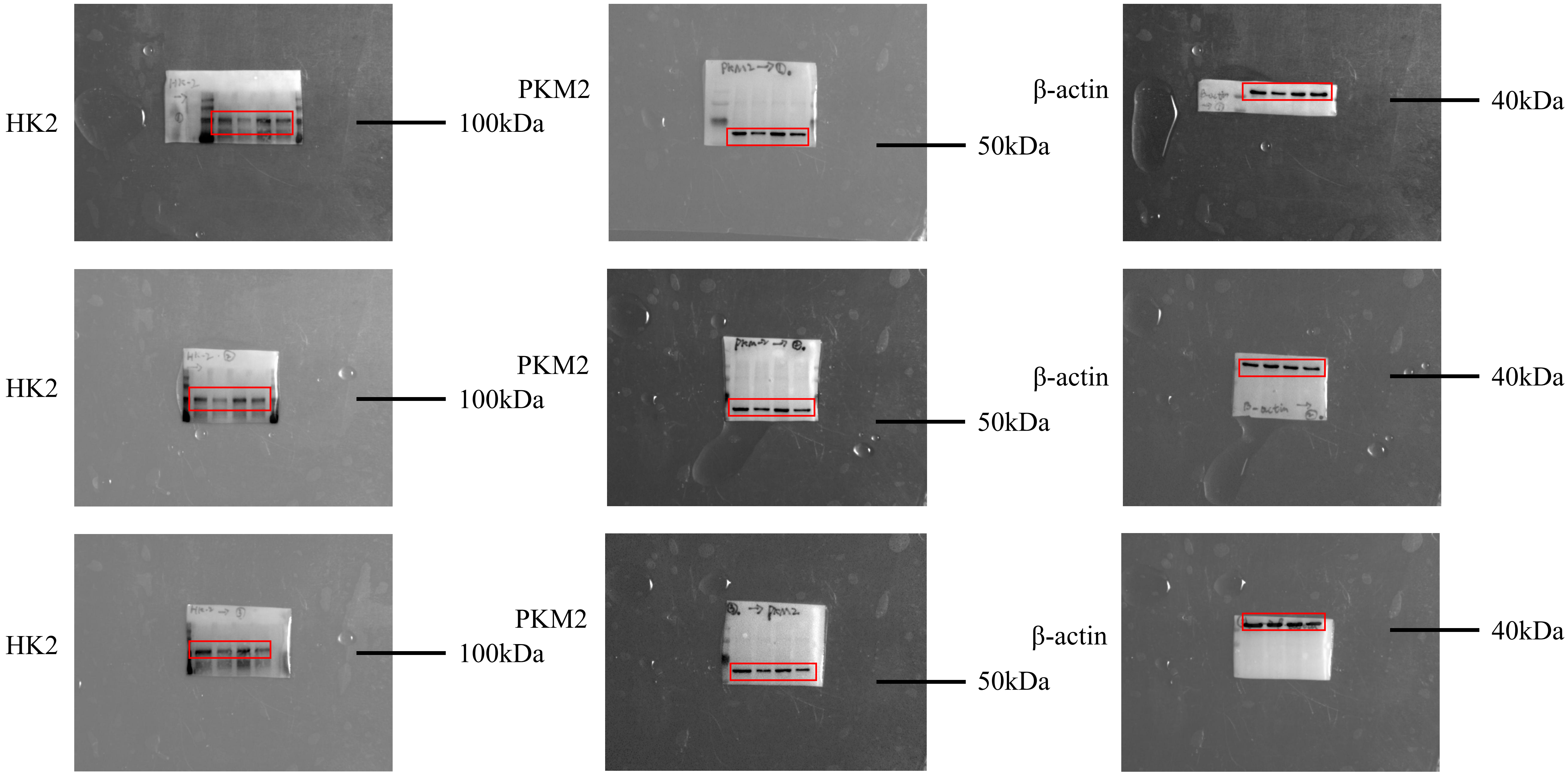

Fig.6f

figure 7

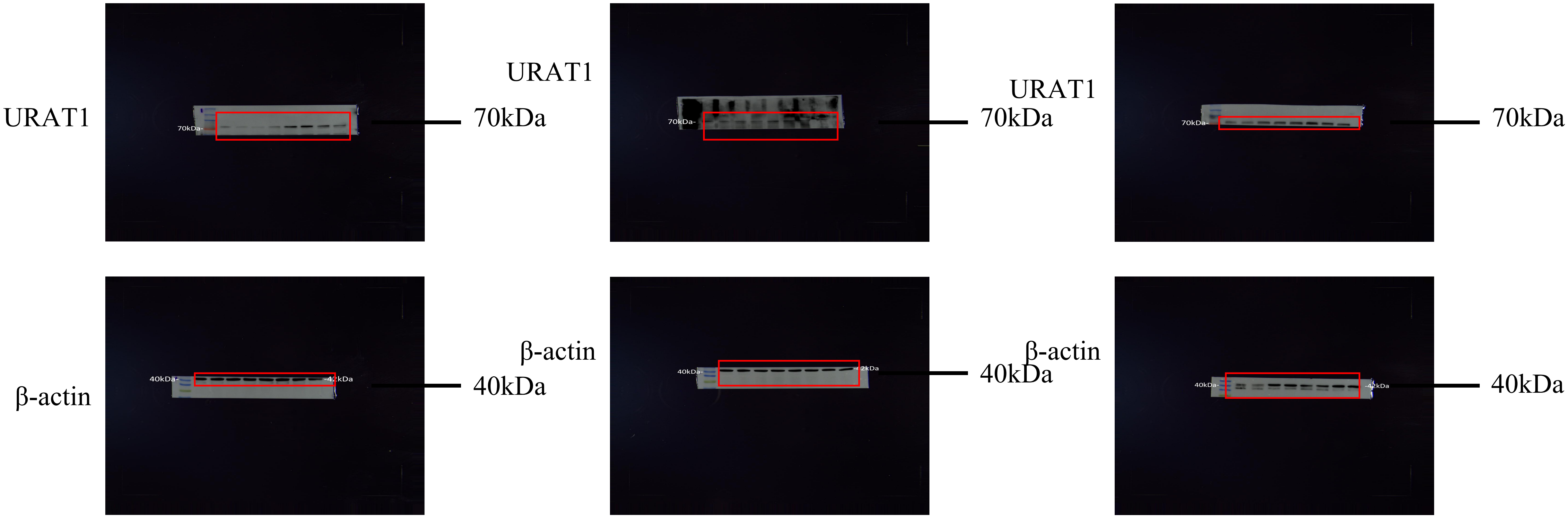

Fig.7h

figure 7

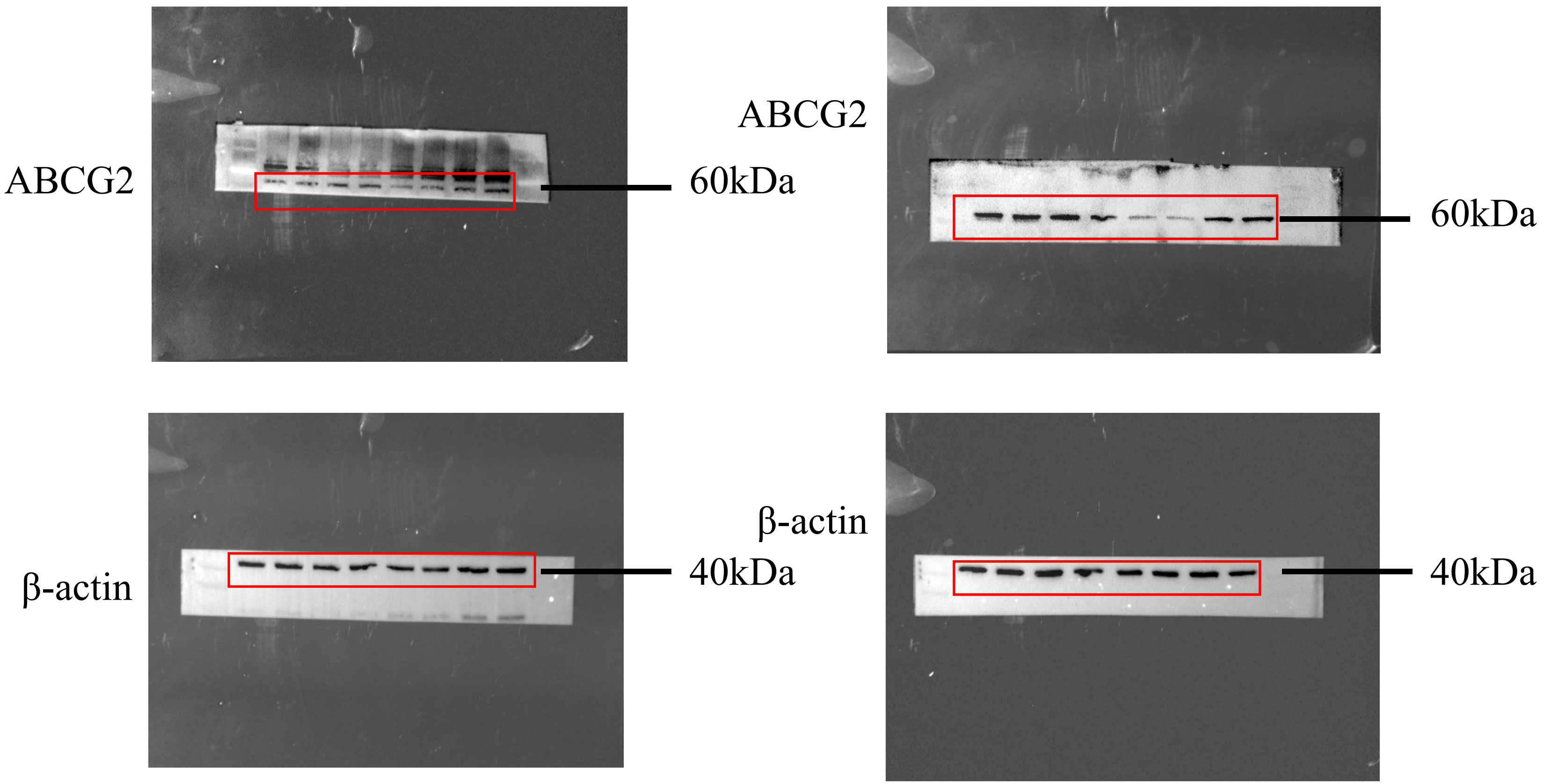

Fig.7h

figure 7

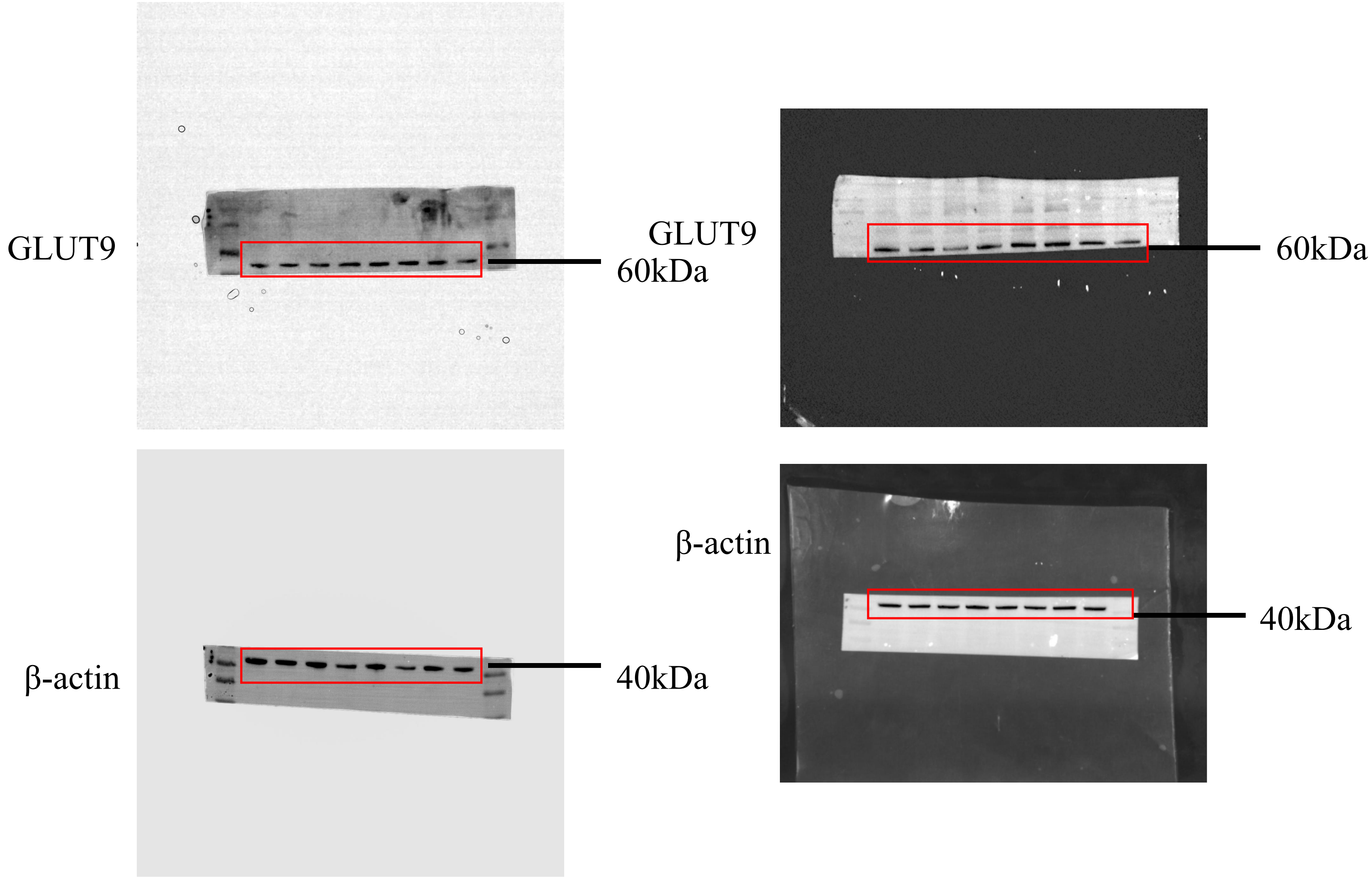

Fig.7h

figure 7

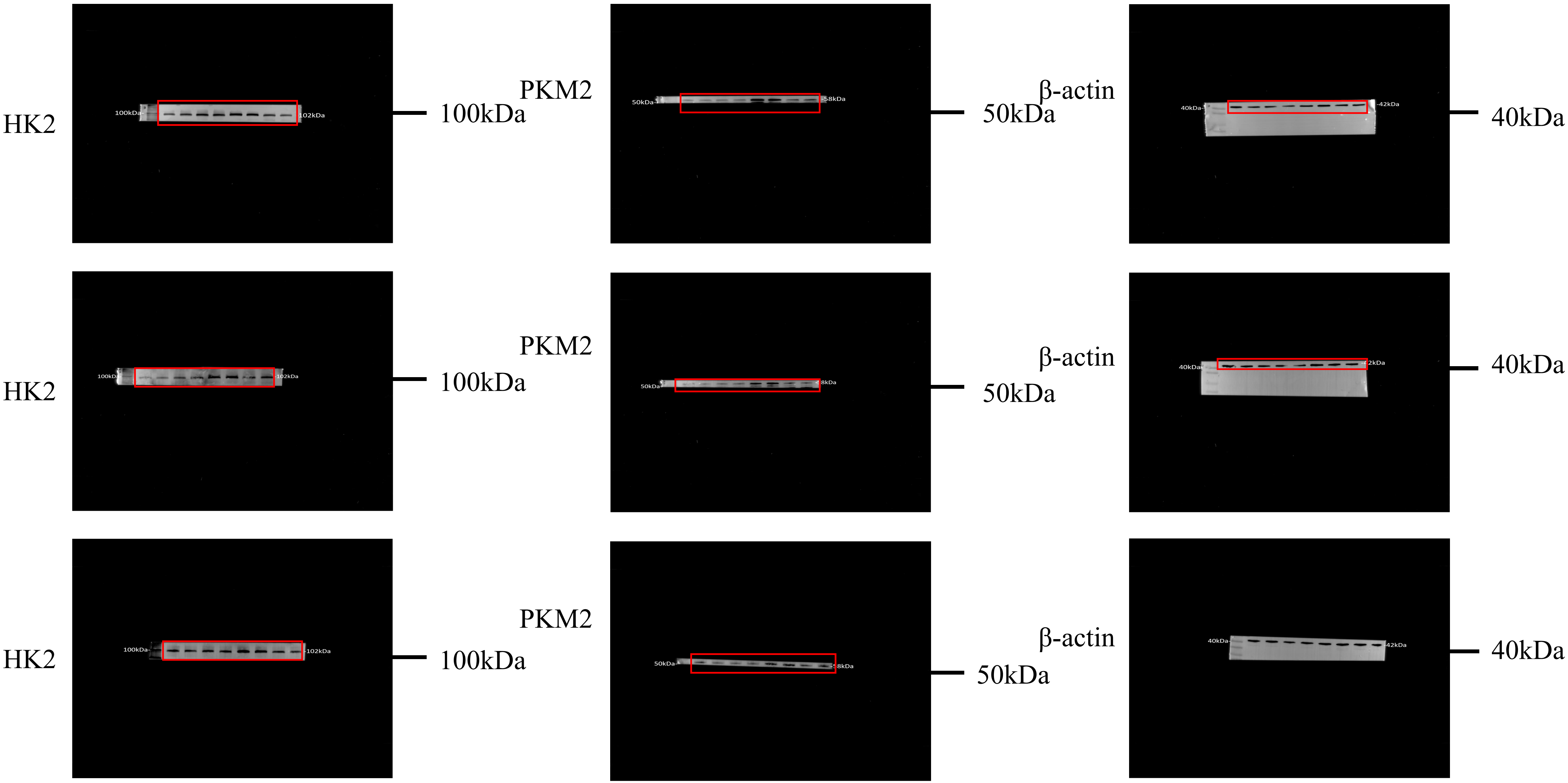

Fig.7h

figure 7

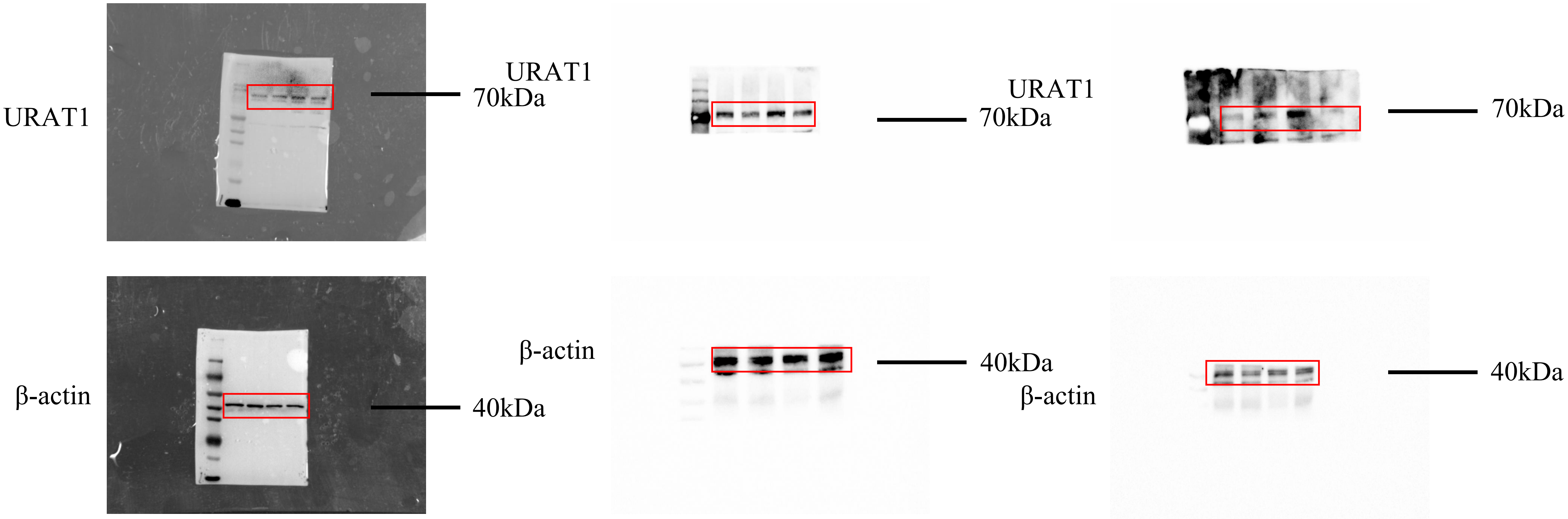

Fig.7o

figure 8

CTSB

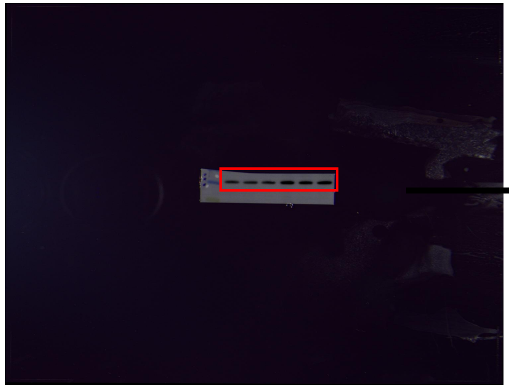

25kDa

β-actin

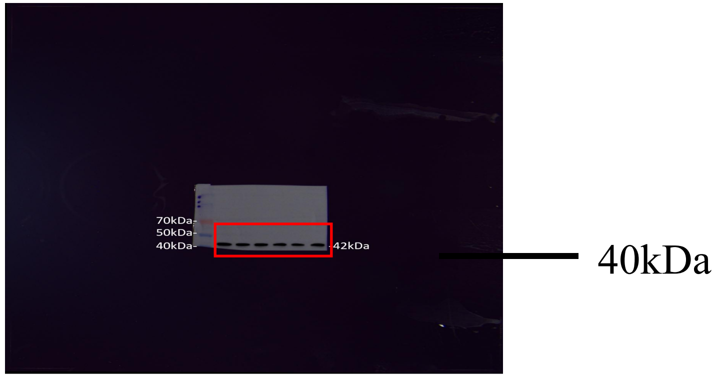

40kDa

Fig.8a

figure 8

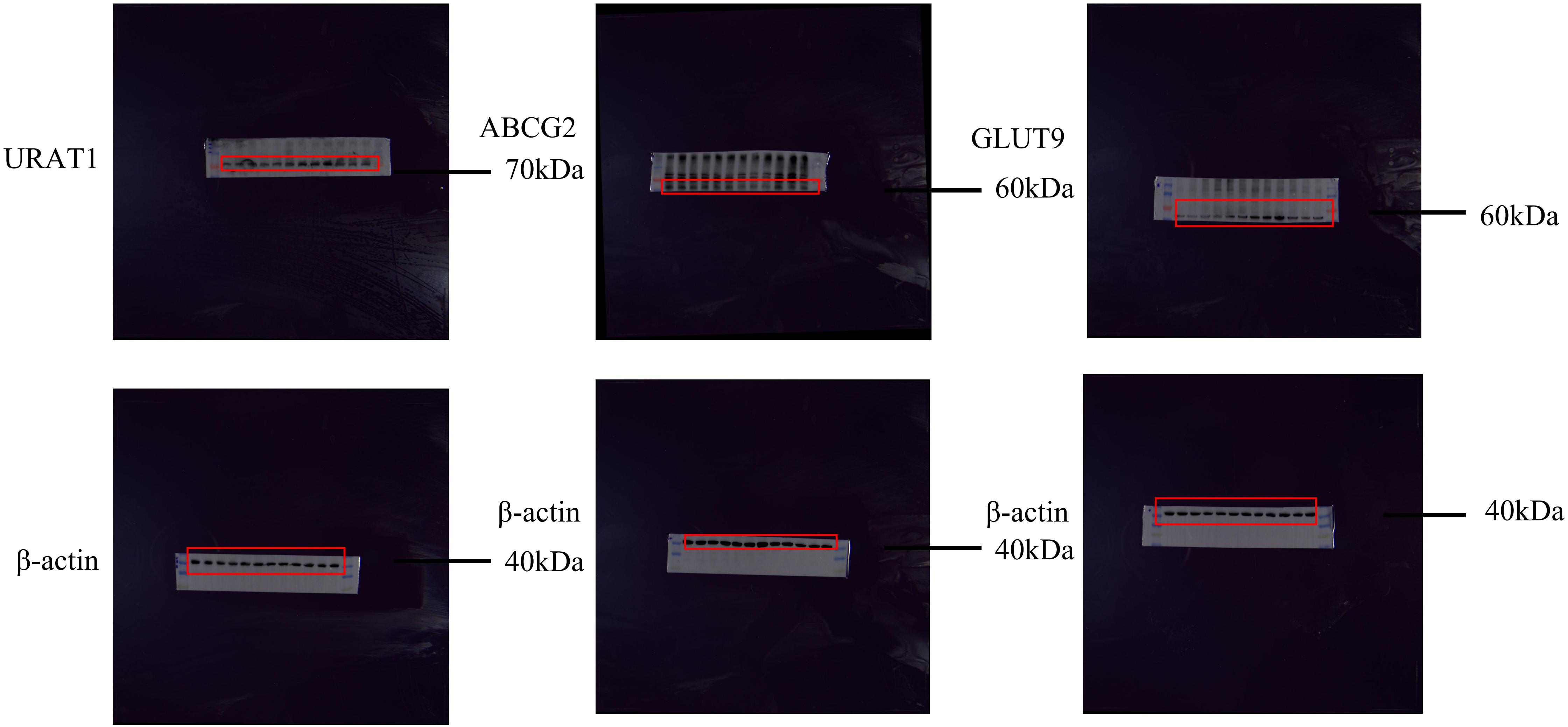

Fig.8c

figure 8

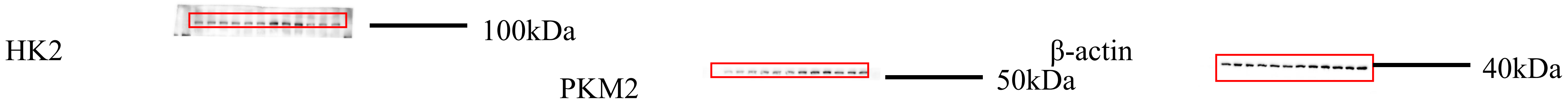

Fig.8g

figure S3

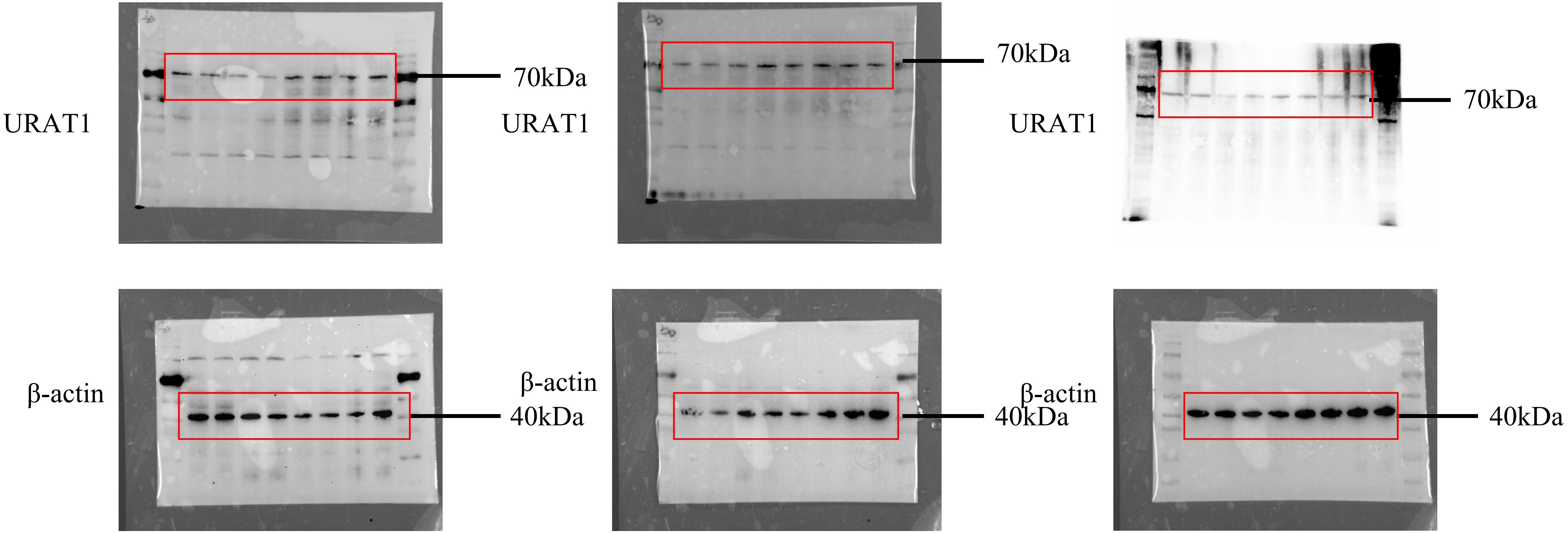

Fig.S3f

figure S3

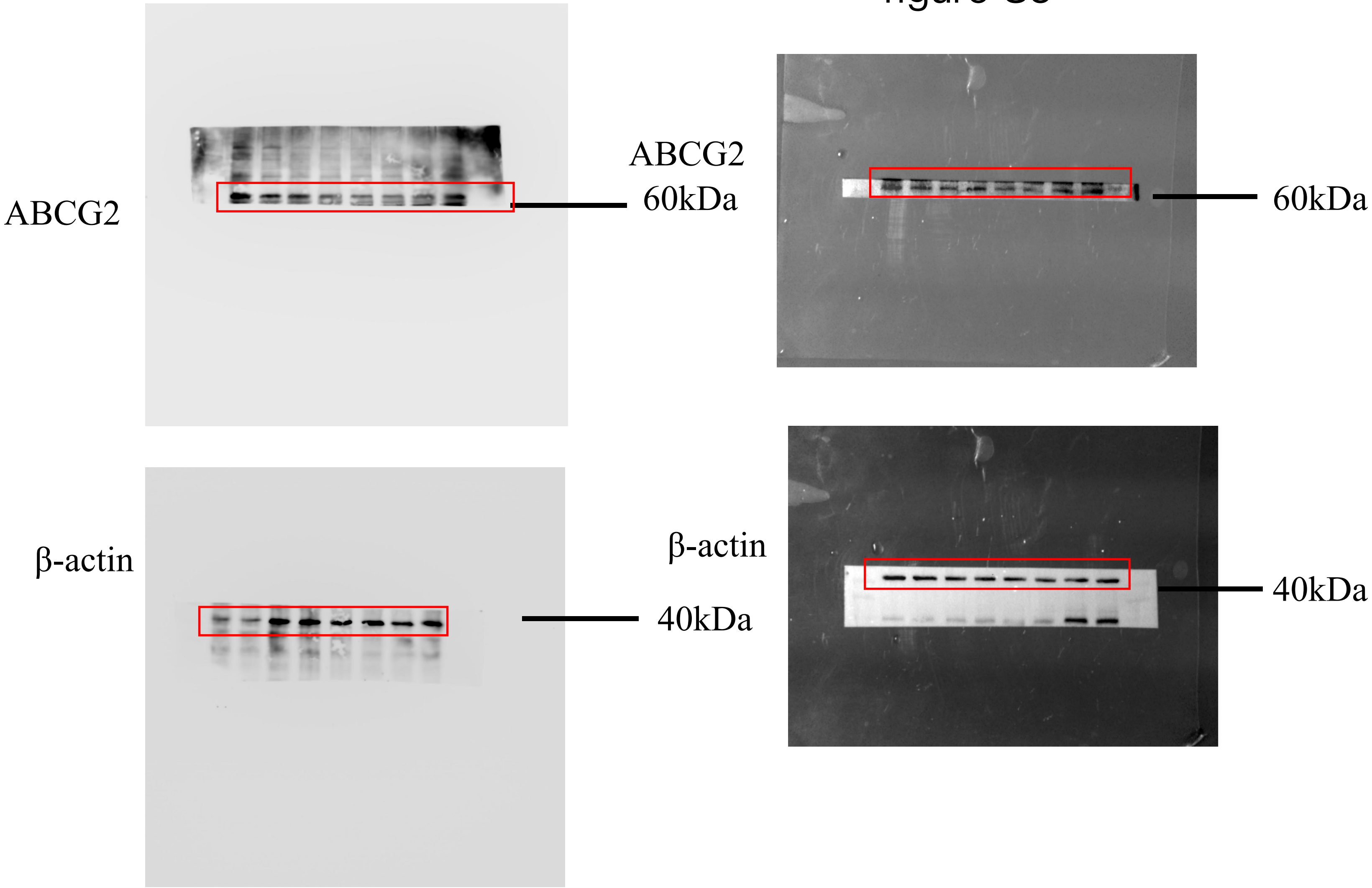

Fig.S3f

figure S3

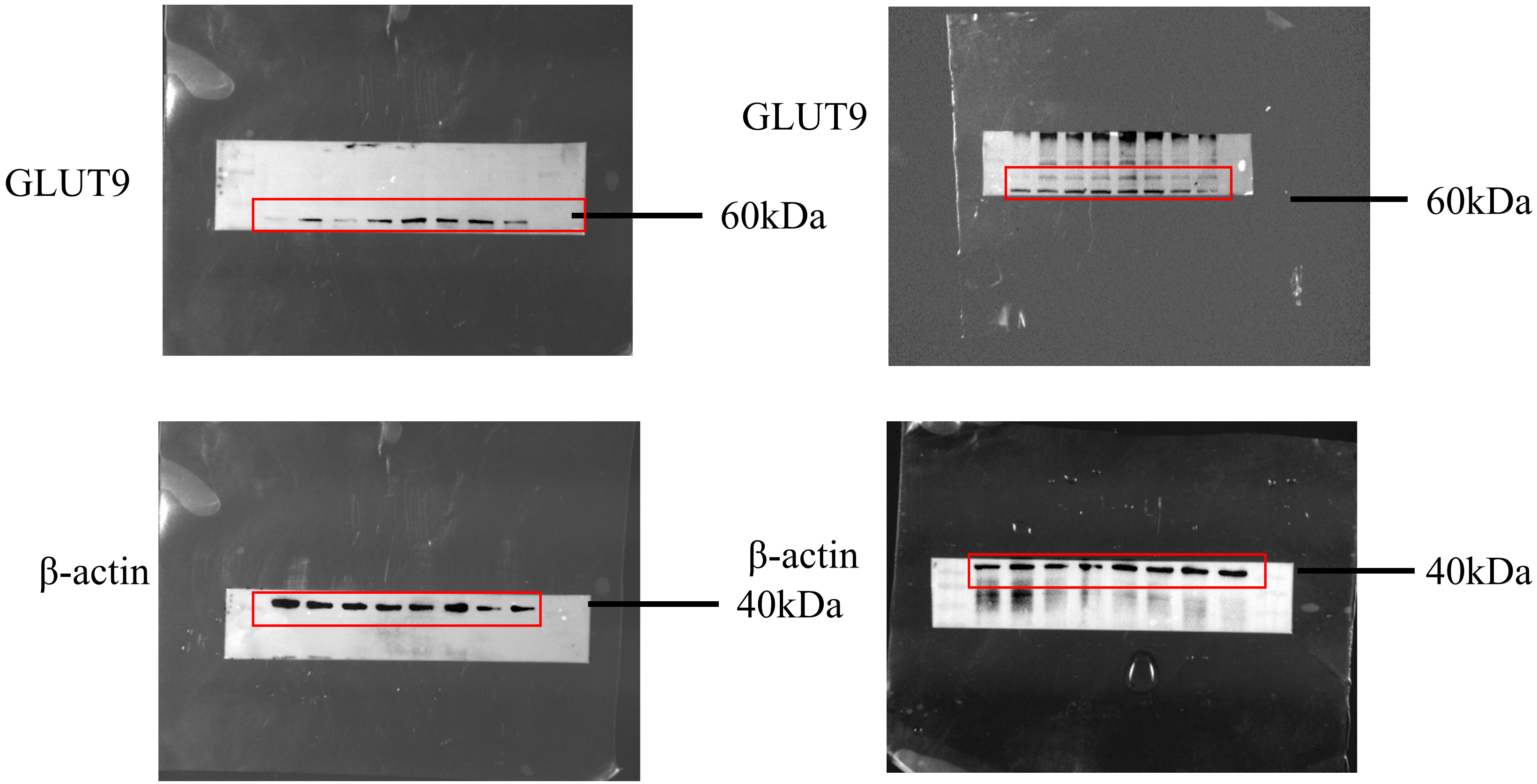

Fig.S3f

figure S4

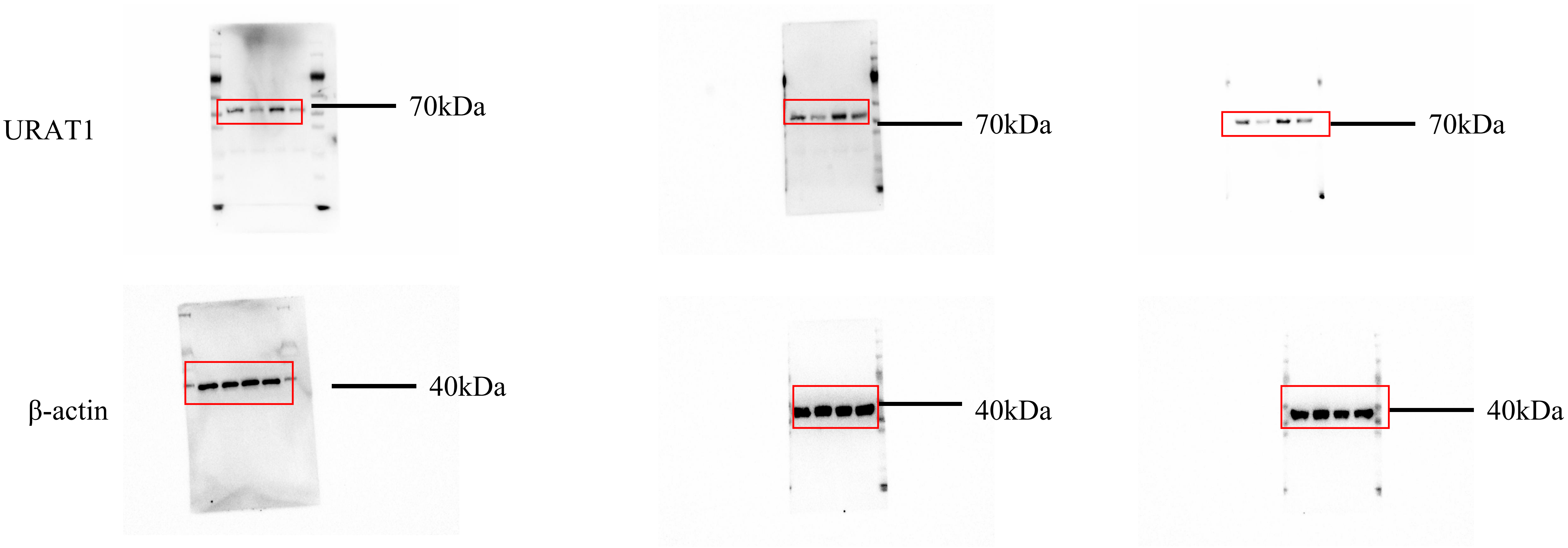

Fig.S4d

figure S4

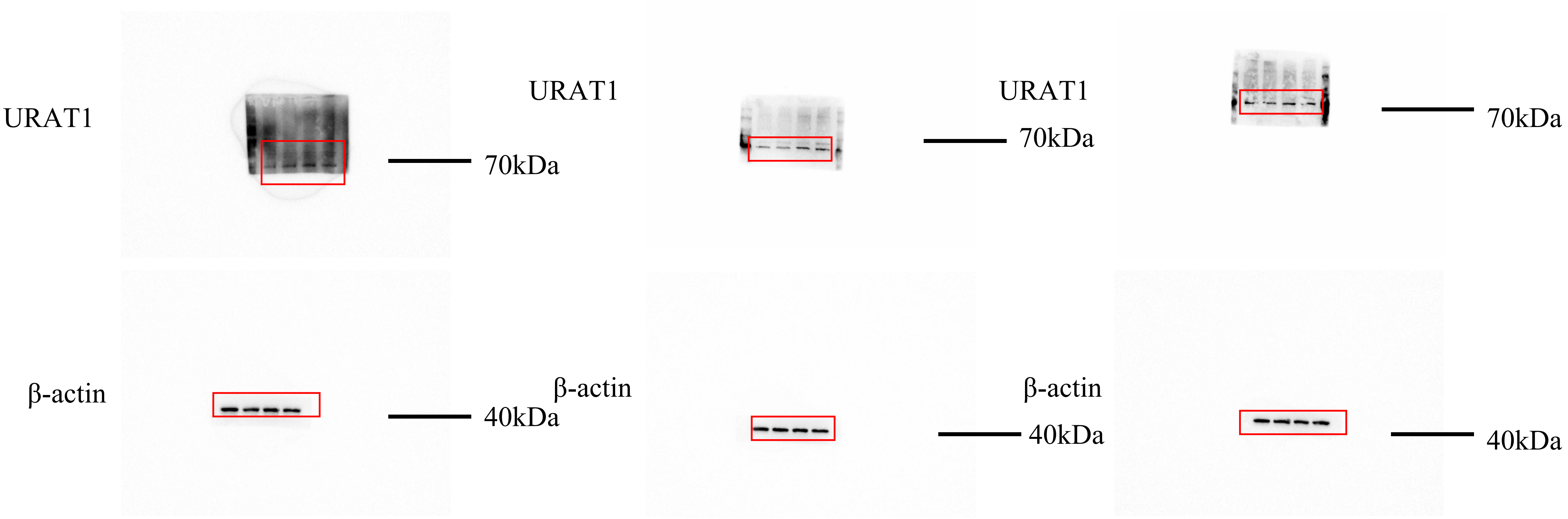

Fig.S4g

figure S6

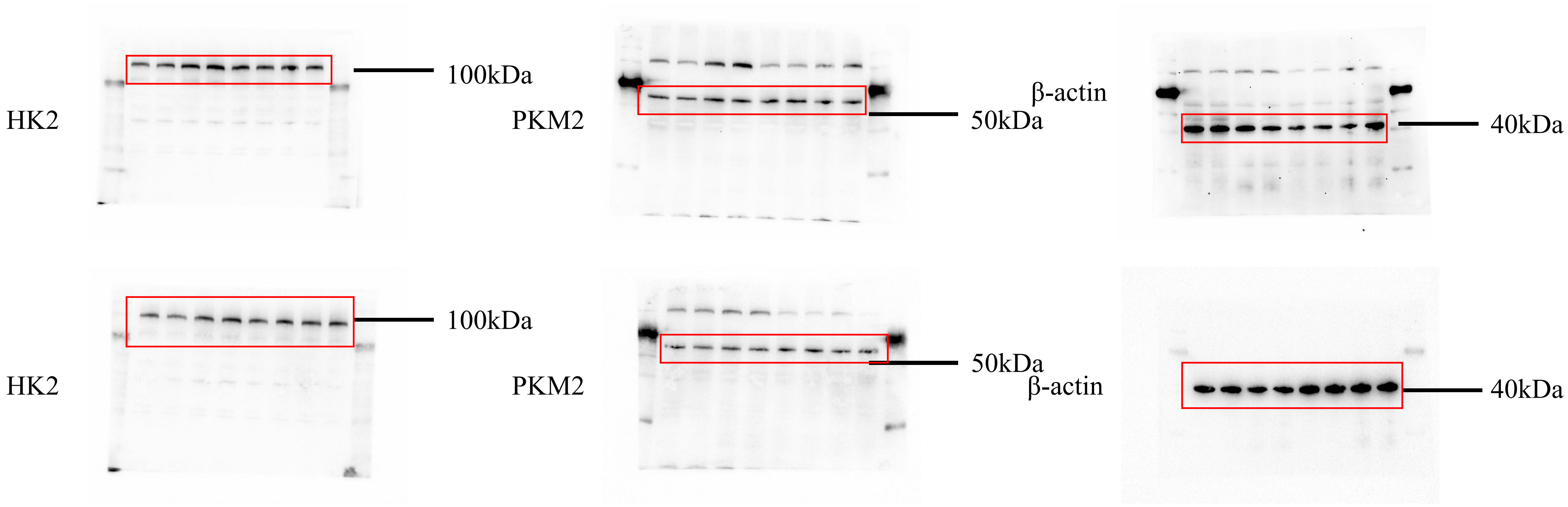

Fig.S6a

figure S6

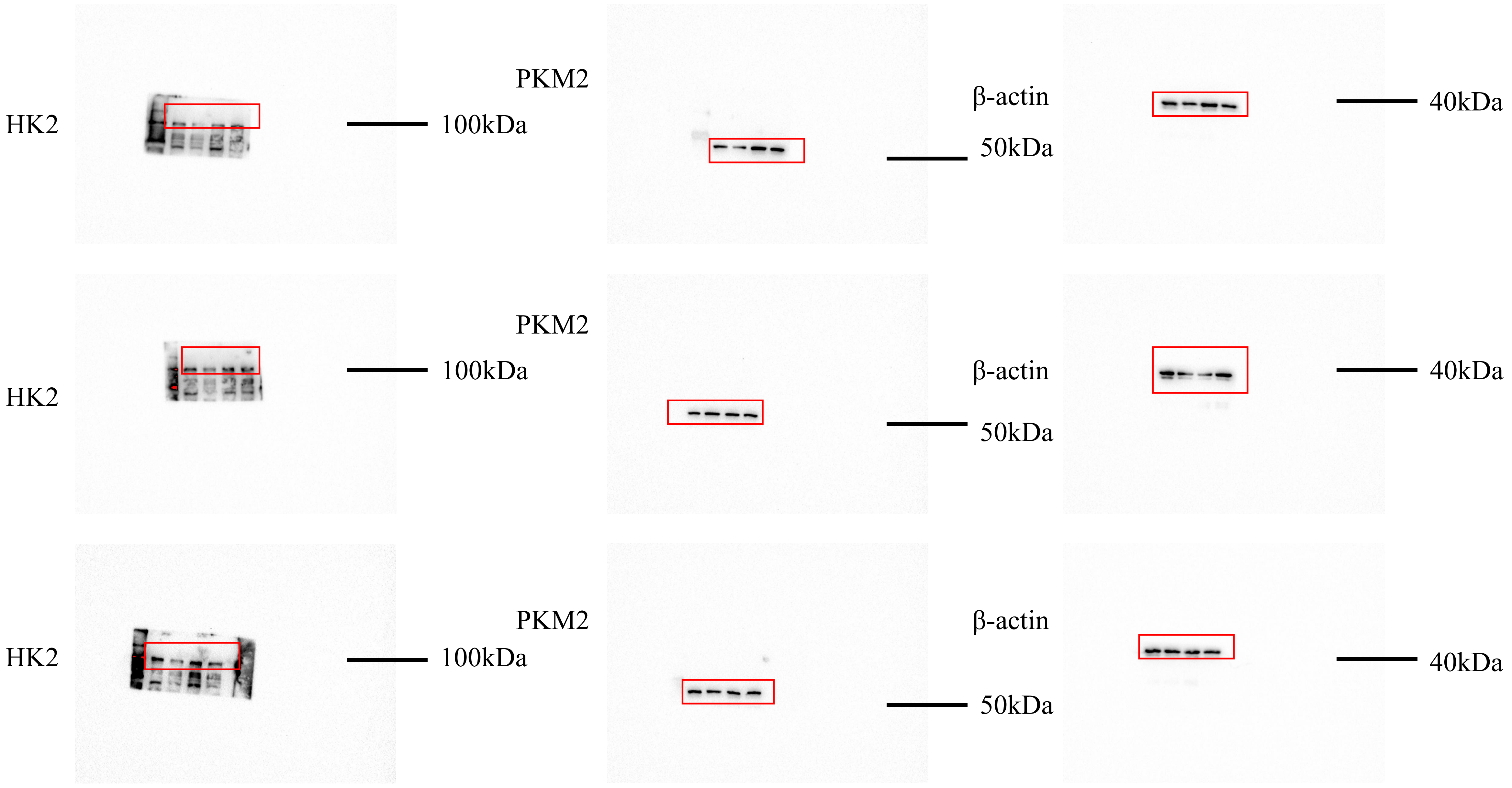

Fig.S6b

Supplementary Figure S7. Uncropped blots.
