# Supplementary material for: Cathepsin B-dependent glycolysis contributes to reduced renal uric acid excretion in hyperuricemia
Source: Commun Biol. 2025 Jun 2;8:845. doi: 10.1038/s42003-025-08303-5 (PMC12130491; doi:10.1038/s42003-025-08303-5)
Supplement: Supplementary file 3 — Description of Additional Supplementary [file 42003_2025_8303_MOESM3_ESM.pdf]

Description of Additional Supplementary Files

File name: Supplementary Data 1
Description: The source data behind the graphs in the paper

File name: Supplementary Data 2
Description: Raw metabolite intensity matrix derived from untargeted LC-MS analysis of mouse kidney tissue samples.
