# Supplementary material for: Cathepsin B-dependent glycolysis contributes to reduced renal uric acid excretion in hyperuricemia
Source: Commun Biol. 2025 Jun 2;8:845. doi: 10.1038/s42003-025-08303-5 (PMC12130491; doi:10.1038/s42003-025-08303-5)
Supplement: Supplementary file 6 — Reporting-summary [file 42003_2025_8303_MOESM6_ESM.pdf]

nature portfolio

Corresponding author(s): Jing Xiao

Last updated by author(s): May 15, 2025

Reporting Summary

Nature Portfolio wishes to improve the reproducibility of the work that we publish. This form provides structure for consistency and transparency in reporting. For further information on Nature Portfolio policies, see our Editorial Policies and the Editorial Policy Checklist.

Statistics

For all statistical analyses, confirm that the following items are present in the figure legend, table legend, main text, or Methods section.

n/a	Confirmed	
☐	☒	The exact sample size (*n*) for each experimental group/condition, given as a discrete number and unit of measurement
☐	☒	A statement on whether measurements were taken from distinct samples or whether the same sample was measured repeatedly
☐	☒	The statistical test(s) used AND whether they are one- or two-sided<br>*Only common tests should be described solely by name; describe more complex techniques in the Methods section.*
☐	☒	A description of all covariates tested
☐	☒	A description of any assumptions or corrections, such as tests of normality and adjustment for multiple comparisons
☐	☒	A full description of the statistical parameters including central tendency (e.g. means) or other basic estimates (e.g. regression coefficient) AND variation (e.g. standard deviation) or associated estimates of uncertainty (e.g. confidence intervals)
☐	☒	For null hypothesis testing, the test statistic (e.g. *F*, *t*, *r*) with confidence intervals, effect sizes, degrees of freedom and *P* value noted<br>*Give P values as exact values whenever suitable.*
☒	☐	For Bayesian analysis, information on the choice of priors and Markov chain Monte Carlo settings
☒	☐	For hierarchical and complex designs, identification of the appropriate level for tests and full reporting of outcomes
☒	☐	Estimates of effect sizes (e.g. Cohen's *d*, Pearson's *r*), indicating how they were calculated

Our web collection on statistics for biologists contains articles on many of the points above.

Software and code

Policy information about availability of computer code

Data collection	spss 26.0
Data analysis	spss 26.0

For manuscripts utilizing custom algorithms or software that are central to the research but not yet described in published literature, software must be made available to editors and reviewers. We strongly encourage code deposition in a community repository (e.g. GitHub). See the Nature Portfolio guidelines for submitting code & software for further information.

Data

Policy information about availability of data

All manuscripts must include a data availability statement. This statement should provide the following information, where applicable:

- Accession codes, unique identifiers, or web links for publicly available datasets
- A description of any restrictions on data availability
- For clinical datasets or third party data, please ensure that the statement adheres to our policy

Raw data will be available upon request by contacting the corresponding author

Research involving human participants, their data, or biological material

Policy information about studies with human participants or human data. See also policy information about sex, gender (identity/presentation), and sexual orientation and race, ethnicity and racism.

Reporting on sex and gender	We selected only males in this study because, according to previous studies, the incidence of hyperuricemia is higher in males than in females
Reporting on race, ethnicity, or other socially relevant groupings	In this study, we used Age, BMI, Scr, eGFR, SUA, FEua and 24-hCTSB as variables, which were selected based on previous literature
Population characteristics	In this study, we used Age, BMI, Scr, eGFR, SUA, FEua and24-hCTSB as variables, which were selected based on previous literature
Recruitment	Hyperuricemia patients who were treated in Huadong Hospital were recruited as subjects, and the basic situation of this study was introduced and informed consent was obtained. After obtaining informed consent and signing the informed consent form, the subjects were screened according to the following procedures within 1 week before the trial: 1) Evaluation of inclusion/exclusion criteria; 2) Demographic data; 3) Vital signs; 4) Inquiry into medical history, recent and current history of combined medication; 5) Clinical laboratory examinations: blood routine, liver and kidney function, urine uric acid, fecal uric acid, blood lipids, glycosylated hemoglobin, etc.
Ethics oversight	Ethics Committee of Huadong Hospital,Fudan University (2022K186).All ethical regulations relevant to human research participants were followed.

Note that full information on the approval of the study protocol must also be provided in the manuscript.

Field-specific reporting

Please select the one below that is the best fit for your research. If you are not sure, read the appropriate sections before making your selection.

☒ Life sciences ☐ Behavioural & social sciences ☐ Ecological, evolutionary & environmental sciences

For a reference copy of the document with all sections, see nature.com/documents/nr-reporting-summary-flat.pdf

Life sciences study design

All studies must disclose on these points even when the disclosure is negative.

Sample size	The sample size was based on previous publication
Data exclusions	No data were excluded from the analyses
Replication	All experiments were repeated at least three times
Randomization	The clinical part is an observational study and does not involve intervention
Blinding	The investigators were blinded to group allocation during data collection and analysis

Reporting for specific materials, systems and methods

We require information from authors about some types of materials, experimental systems and methods used in many studies. Here, indicate whether each material, system or method listed is relevant to your study. If you are not sure if a list item applies to your research, read the appropriate section before selecting a response.

Materials & experimental systems

n/a	Involved in the study	
☐	☒	Antibodies
☐	☒	Eukaryotic cell lines
☒	☐	Palaeontology and archaeology
☐	☒	Animals and other organisms
☐	☒	Clinical data
☒	☐	Dual use research of concern
☒	☐	Plants

Methods

n/a	Involved in the study	
☒	☐	ChIP-seq
☒	☐	Flow cytometry
☒	☐	MRI-based neuroimaging

Antibodies

Antibodies used	Anti-CTSB (# 31718S), anti-rabbit IgG, HRP-linked antibody (# 7074), and anti-mouse IgG, HRP-linked antibody (# 7076), were purchased from CST (Boston, USA). Anti-HK2 (# 66974-1-Ig), anti-PKM2 (# 15822-1-AP), anti-URAT1 (# 14937-1-AP) anti-ABCG2 (# 27286-1-AP) and anti-GLUT9 (# 67530-1-Ig) antibodies were purchased from Proteintech (Chicago, USA).Any other antibodies used can be found in the manuscript
Validation	The validation of the primary antibody can be found on the supplier's website based on the antibody information provided in the article

Eukaryotic cell lines

Policy information about cell lines and Sex and Gender in Research

Cell line source(s)	Human renal proximal tubular epithelial cells (HK-2) (Cyagen Biosciences, Suzhou, China)<br>Mouse renal tubular epithelial cells (MRTECs) (Meisen, China)
Authentication	All cells have been verified by the company
Mycoplasma contamination	All cells are free of mycoplasma contamination
Commonly misidentified lines<br>(See ICLAC register)	HK2,MRTEC

Animals and other research organisms

Policy information about studies involving animals; ARRIVE guidelines recommended for reporting animal research, and Sex and Gender in Research

Laboratory animals	C57BL/6, 8 weeks
Wild animals	The study did not involve wild animals
Reporting on sex	We selected only males in this study because, according to previous studies, the incidence of hyperuricemia is higher in males than in females
Field-collected samples	Mice were housed at room temperature (22-25°C) under standard conditions: humidity of 40-60%, allowing free access to water and food under a 12 h light/dark cycle
Ethics oversight	Ethics Committee of Animal Experiments of Fudan university(2023-HDYY-74JZS)

Note that full information on the approval of the study protocol must also be provided in the manuscript.

Clinical data

Policy information about clinical studies

All manuscripts should comply with the ICMJE guidelines for publication of clinical research and a completed CONSORT checklist must be included with all submissions.

Clinical trial registration	Registered in Chinese Clinical Trial Registry with the registration number of ChiCTR2200065733
Study protocol	Available from the corresponding author
Data collection	Urine samples from 24 adult male patients were collected over 24 hours into a clean plastic bucket with a lid. In 24-hour urine collection, the first morning void is discarded and all urine is collected for the next 24 hours, including the first morning void the next day
Outcomes	According to our experimental purpose, we mainly want to observe the changes in urine CTSB in different groups

Plants

Seed stocks	N/A
Novel plant genotypes	N/A
Authentication	N/A
